# The location and translocation of *ndh* genes of chloroplast origin in the Orchidaceae family

**DOI:** 10.1038/srep09040

**Published:** 2015-03-12

**Authors:** Choun-Sea Lin, Jeremy J. W. Chen, Yao-Ting Huang, Ming-Tsair Chan, Henry Daniell, Wan-Jung Chang, Chen-Tran Hsu, De-Chih Liao, Fu-Huei Wu, Sheng-Yi Lin, Chen-Fu Liao, Michael K. Deyholos, Gane Ka-Shu Wong, Victor A. Albert, Ming-Lun Chou, Chun-Yi Chen, Ming-Che Shih

**Affiliations:** 1 Agricultural Biotechnology Research Center, Academia Sinica, Taipei, Taiwan; 2 Institute of Biomedical Sciences, National Chung-Hsing University, Taichung, Taiwan; 3 Department of Computer Science and Information Engineering, National Chung Cheng University, Chiayi, Taiwan; 4 Academia Sinica Biotechnology Center in Southern Taiwan, Tainan, Taiwan; 5 Departments of Biochemistry and Pathology, University of Pennsylvania School of Dental Medicine, Philadelphia, PA, USA; 6 Department of Biological Sciences, University of Alberta, Edmonton, AB, Canada; 7 Department of Medicine, University of Alberta, Edmonton AB, Canada; 8 BGI-Shenzhen, Beishan Industrial Zone, Yantian District, Shenzhen, China; 9 Department of Biological Sciences, University at Buffalo, Buffalo, NY, USA; 10 Department of Life Sciences, Tzu Chi University, Hualien, Taiwan

## Abstract

The NAD(P)H dehydrogenase complex is encoded by 11 *ndh* genes in plant chloroplast (cp) genomes. However, *ndh* genes are truncated or deleted in some autotrophic Epidendroideae orchid cp genomes. To determine the evolutionary timing of the gene deletions and the genomic locations of the various *ndh* genes in orchids, the cp genomes of *Vanilla planifolia*, *Paphiopedilum armeniacum*, *Paphiopedilum niveum*, *Cypripedium formosanum*, *Habenaria longidenticulata*, *Goodyera fumata* and *Masdevallia picturata* were sequenced; these genomes represent Vanilloideae, Cypripedioideae, Orchidoideae and Epidendroideae subfamilies. Four orchid cp genome sequences were found to contain a complete set of *ndh* genes. In other genomes, *ndh* deletions did not correlate to known taxonomic or evolutionary relationships and deletions occurred independently after the orchid family split into different subfamilies. In orchids lacking cp encoded *ndh* genes, non cp localized *ndh* sequences were identified. In *Erycina pusilla*, at least 10 truncated *ndh* gene fragments were found transferred to the mitochondrial (mt) genome. The phenomenon of orchid *ndh* transfer to the mt genome existed in *ndh*-deleted orchids and also in *ndh* containing species.

Eukaryotic cells arose through the engulfment of bacterial endosymbionts and the subsequent gradual conversion of those bacteria into organelles (mitochondria and chloroplasts)^1^,^2^. During this process, there was a massive transfer of genes from the endosymbiont genomes into the nuclear genome of the host cell^3^. However, plant chloroplasts (cp) possess their own genomes, which contain genes that are involved in photosynthesis, transcription and translation^4^. The numbers and functions of these cp genes are highly conserved among higher plants. One family of genes that is involved in photosynthesis is the *ndh* family, of which 11 members encode NADH dehydrogenase subunits. These genes are homologs of those encoding mitochondrial NADH dehydrogenase subunits, which are involved in respiratory electron transport^5^,^6^. In angiosperm chloroplasts, these ndh proteins associate with nuclear-encoded subunits to form the NADH dehydrogenase-like complex. This protein complex associates with photosystem I to become a super-complex that mediates cyclic electron transport^7^, produces ATP to balance the ATP/NADPH ratio, and facilitates chlororespiration when cyclic electron transport pauses overnight^8^.

Heterotrophic plants, which do not photosynthesize, lack functional *ndh* genes^9^. Interestingly, some autotrophic plants, such as pines, Gnetales, *Erodium*, *Melianthus* and orchids also lack functional *ndh* genes in their cp genomes^10^,^11^,^12^,^13^,^14^,^15^,^16^,^17^,^18^. There are five subfamilies in Orchidaceae: Apostasioideae, Vanilloideae, Cypripedioideae, Orchidoideae and Epidendroideae^19^,^20^,^21^. Chloroplast genomes from four autotrophic Epidendroideae orchid genera, *Phalaenopsis*, *Oncidium*, *Erycina*, and *Cymbidium*, have been sequenced and are publicly available^11^,^14^,^16^,^17^. From these orchid cp genomes, only *ndhB* of *Oncidium* ‘Gower Ramsey' and *ndhE*, *J*, and *C* of *Cymbidium* have been predicted to encode functional *ndh* proteins^14^,^17^. All the other *ndh* gene fragments contain nonsense mutations or are truncated or absent from the plastome^11^,^14^,^16^,^17^. For example, the cp genome of *E. pusilla* contains truncated versions of *ndhJ*, *C*, *D*, *B*, *G*, and *H* and lacks sequences for *ndhK*, *F*, *E*, *A*, and *I*^16^. These results indicate that deletion and truncation of *ndh* gene fragments are common to orchid cp genomes. However, the *ndh* genes in the cp genome of *Apostasia wallichii* (Apostasioideae) are transcribed and are predicted to encode functional proteins^22^. These findings indicate that the orchid common ancestor contained an entire functional set of *ndh* genes. So far only Epidendroideae cp genome sequences have been published^11^,^14^,^16^,^17^. Therefore, understanding which orchid subfamilies lack *ndh* genes in their cp genomes has the potential to considerably increase understanding of photosynthetic evolution in orchids.

Previous studies have demonstrated that plant cpDNA has been transferred to both the mitochondrial (mt)^23^,^24^ and nuclear genomes^1^,^25^,^26^,^27^ Based on PCR results, Chang et al.^11^ proposed that in *Phalaenopsis*, the ancestral *ndh* genes have been transferred to the genome of the nucleus or another organelle. Recently published mitochondrial genomes from different seed plant species contain 1 to 10% cpDNA originating from various regions of the plastome^28^,^29^,^30^. Due to the size and complexity of orchid genomes, it is difficult to obtain these sequences by direct sequencing or genome walking^31^. To resolve this problem, we previously employed a well-established strategy, using simple PCR to identify BAC clones containing sought-after genes in both *E. pusilla* and *O.* ‘Gower Ramsey'^16^,^31^. We also used this strategy to identify and sequence BAC libraries that contained cp genomic fragments, which resulted in the sequencing of the complete *E. pusilla* cp genome^16^. This targeted method was more cost- and time-effective than whole genome sequencing.

In this report, the evolutionary timings of *ndh* deletions from orchid cp genomes was investigated. The cp genomes of *Vanilla planifolia* (Vanilloideae), *Paphiopedilum armeniacum*, *Paphiopedilum niveum*, *Cypripedium formosanum* (Cypripedioideae), *Habenaria longidenticulata*, *Goodyera fumata* (Orchidoideae), and *Masdevallia picturata* (Epidendroideae) were sequenced. In addition, the mitochondrial locations of the *ndh* genes were investigated in *E. pusilla*, an Epidendroideae model orchid, by sequencing BAC clones that contained the *ndh* genes that were missing from the cp genome.

## Results

### *ndh* genes among the orchid subfamilies

There are 11 *ndh* genes in higher plant chloroplast genomes. To understand the differential expression of these genes among the orchid subfamilies, *ndh* transcripts were identified in 16 species from five Orchidaceae subfamilies (Supplementary Table S1 and S2). *Neuwiedia malipoensis* (Apostasioideae), *Cypripedium singchii* (Cypripedioideae), *Habenaria delavayi*, *Goodyera pubescens* (Orchidoideae), *Masdevallia yuangensis* and *Cymbidium sinense* (Epidendroideae) had all 11 *ndh* genes. However, *Vanilla shenzhenica, V. planifolia, Galeola faberi* (Vanilloideae)*, Drakaea elastica* (Orchidoideae), and *E. pusilla* (Epidendroideae), had only less 5 *ndh* gene sequences (Supplementary Table S1). The number of *ndh* gene sequences in each transcriptome did not correlate with known orchid evolutionary relationships (Supplementary Table S1). The fewest *ndh* transcripts were found in Vanilloideae. Previous studies have reported *ndh* deletions in Epidendroideae species, but an analysis of the *M. yuangensis* transcriptome database revealed highly conserved sequences (79.4%) for all of the *ndh* genes. The same phenomenon also occurred in *C. sinense* (Supplementary Table S1). These results indicate that the loss of *ndh* genes from the orchid species occurred independently within the subfamilies.

**Figure 1:**
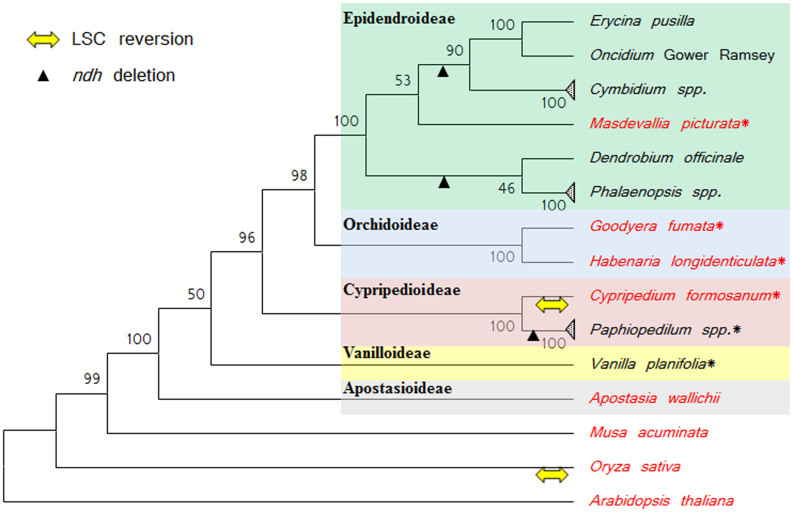
Phylogenetic analysis of orchids. The phylogenetic tree was based on the chloroplast *rbcL*, *matK*, *psaA*, *psaB*, and *rpoC2* nucleotide sequences. The species names in red are *ndh*-complete and in black are *ndh*-deleted genomes. The asterisk (*) indicates that the cp genomes were sequenced in this report. The percentages of replicate trees in which the associated taxa clustered together in the bootstrap test (1000 replicates) are shown next to the branches.

**Figure 2:**
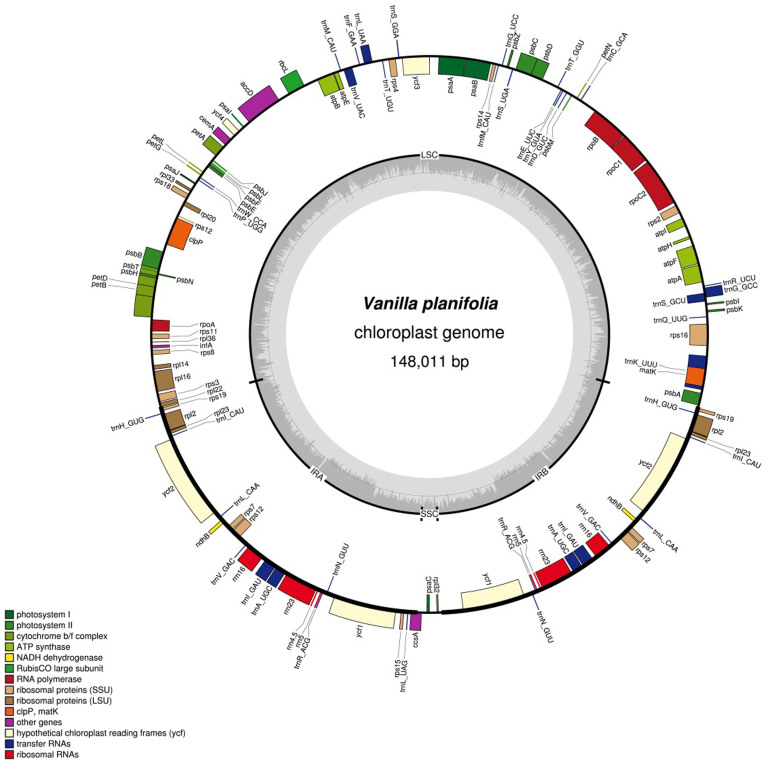
Gene maps of *Vanilla planifolia* chloroplast genomes. Genes on the outside of the map are transcribed clockwise whereas genes on the inside of the map are transcribed counterclockwise. Colors indicate genes with different functional groups.

The cp genomes of seven orchids in four subfamilies were determined (Figure 1 and 2, Supplementary Figure S1, Supplementary Tables S3 and S4). The read coverage of the seven assembled chloroplast genomes is shown in Supplementary Table S5. When all 11 *ndh* DNAs in the cp genome were in frame and encoded the full-length *ndh* gene, the genomes are classified as *ndh*-complete type cp genome. If any one of the 11 *ndh* genes had nonsense mutations, deletions, or was absent, the genomes are classified as *ndh-*deleted type cp genome. There were both *ndh*-complete- and *ndh*-deleted types in Cypripedioideae [*Paphiopedilum armeniacum* (*ndh*-deleted), *Paphiopedilum niveum* (*ndh*-deleted), and *C. formosanum* (*ndh*-complete)] and in Epidendroideae [*E. pusilla* (*ndh*-deleted) and *M. picturata* (*ndh*-complete)]. Among the only Vanilloideae species investigated, *V. planifolia*, was an *ndh*-deleted type, but this result cannot exclude the possibility of *ndh-*complete species in other Vanilloideae species. The two sequenced cp genomes in Orchidoideae, *H. longidenticulata* and *G. fumata*, are both *ndh*-complete. Based on the transcriptome results, *D. elastica*, an endangered native Australian orchid, has few *ndh* transcripts (Supplementary Table S1). This species may be an example of *ndh-*deleted Orchidoideae.

**Figure 3:**
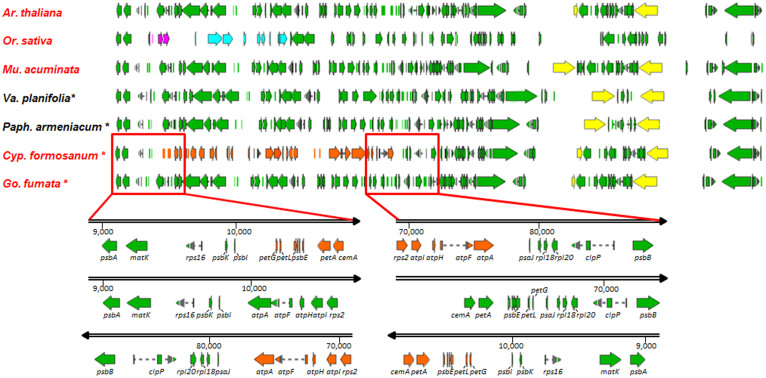
Genome order of the chloroplast-coding regions in 4 orchid genera, *Arabidopsis thaliana*, *Oryza sativa*, and *Musa acuminata*. The name in red indicates that the cp genome contains complete *ndh* genes. Black indicates that the *ndh* genes are deleted. The asterisk (*) indicates the cp genomes were sequenced in this study. *P. niveum* and *P. armeniacum* have the same genome structure and order. *H. longidenticulata*, *M. picturata*, and *G. fumata* have the same genome structure and gene order. The arrowheads indicate the direction of the genes. The pink arrows indicate that the rice *psbC* and *psbD* are inserted next to *psbI* but are located behind *psbM* in the other plant cp genomes. Blue indicates that the sequence region from *atpA* to *psbM* is reversed in the rice cp genome. The yellow arrow indicates that there is a full-length *ycf1* gene in the IR region. Orange indicates that the *atpA-petG* region is reversed in *C. formosanum*. The lower panels are a zoom-in of the box regions. The arrowheads indicate the direction of sequences from 5′ to 3′. The numbers indicate the position in the cp genome.

In addition to the *ndh* gene profiles, other differences were found between these seven orchid cp genomes (Figure 3). Notably, the region from *atpA* to *petG* is reversed in the *C. formosanum* cp genome. These results indicate that the cp genomes of these seven orchids provide useful information on *ndh* diversity in the family.

### Identification of non-cp *ndh* genes in orchids

A comparison of the cp genomic and transcriptomic data showed that only *ndhB* was found in the *V. planifolia* cp genome, whereas *ndhK*, *J,* and *C* transcripts were identified in the *V. planifolia* whole-cell transcriptome. Likewise, in the *P. armeniacum* cp genome, only the *ndhD* sequence was encoded in the *ndhF-D-E-G-I-A-H* region of the cp genome. However, the *ndhA* and *ndhH* transcripts were present in the *P. armeniacum* whole-cell transcriptome, indicating that *ndhA* and *ndhH* gene were encoded in either the mt or nuclear genomes in *P. armeniacum* (Supplementary Table S6). These results, therefore, indicate that non-cp *ndh* genes exist and may be transcribed. We therefore attempted to identify these genes in mt or nuclear genome sequences.

Many non-cp *ndh* gene fragments were identified in the orchid whole genomic sequences. In addition to the *ndh*-deleted species, the *ndh*-complete species (*C. formosanum*, *H. longidenticulata*, *G. fumata*, and *M. picturata*) also contained non-cp *ndh* gene fragments (Supplementary Tables S3 and S4). Because *ndh* genes in the cp genomes of all plant species are clustered as *ndhJ-K-C*, *ndhF-D-E-G-I-A-H* and *ndhB*, the results below are presented in this order.

**Figure 4:**
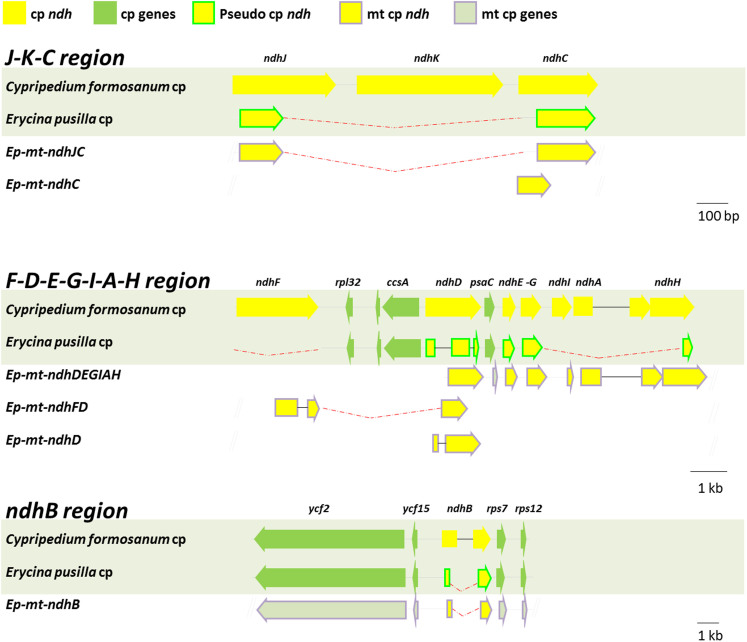
Comparison of the *Cypripedium formosanum* cp, *Erycina pusilla* cp genomes, and the mt *ndh* regions. The arrows indicate coding regions. The arrowheads indicate the direction of the genes.

Although flanking DNA sequences can provide information to identify the organelle from which they were derived, some of the sequences were too similar to cp genome sequences or were too short to identify (Supplementary Table S4, Paph_niveum_ndhB1 to Mas_ndhJK). Therefore, it would be important to extend the sequence length in future studies. In recent studies, *E. pusilla* has been established as a model for orchid genome research^16^. Using *ndh*-containing sequences, which were derived from the genomic NGS sequences, specific primers were designed to screen the *E. pusilla* BAC library^16^. The BAC clones were identified and sequenced to confirm presence of *ndh*-like gene fragments and to determine the organelle of origin. Six BAC clones that contained putative *ndh* gene fragments were identified: *Ep-mt-ndhJC*, *Ep-mt-ndhC*, *Ep-mt-ndhDEGIAH*, *Ep-mt-ndhDF*, *Ep-mt-ndhD* and *Ep-mt-ndhB* (Figure 4). An analysis of the flanking sequences within the BACs revealed that all six of the contigs were derived from the mt genome. According to genome sequencing data, nine *ndh* genes were transferred to the mt genome in *M. picturata*, eight in *P. niveum* and *G. fumata*, six in *P. armeniacum*, and four in *V. planifolia* (Supplementary Table S3, Supplementary Figures S2, S3, and S4). In total, *E. pusilla* had the greatest number of *ndh* gene fragments transferred to the mt genome (10 genes in six fragments, Figure 4, Supplementary Table S3). Furthermore, there were eight *ndh* genes in *G. fumata* and nine in *M. picturata*, whose cp genomes contained a complete *ndh* profile (Supplementary Table S3). These results showed that the number of transferred *ndh* gene fragments did not correlate with *ndh* deletions in the cp genome.

To understand structural differences between the *mt-ndh* regions and the original cp region, *E. pusilla* mt genome sequence was aligned with the corresponding *C. formosanum* (*ndh*-complete) and *E. pusilla* cp genome regions. This alignment showed a massive loss of DNA in the *ndh* copies that were transferred to the *E. pusilla* mt genome (Figure 4, red-dotted lines). It is also clear that all of the *mt-ndh* fragments were truncated or contained large deletions. Interestingly, some of the *ndh* transfers may have occurred after an *ndh* deletion within the cp genome (such as *Ep-mt-ndhJC*, Figure 4). Alternatively, the complete *ndhJ-K-C* region may have first been transferred to the mt genome and then undergone deletions.

### Relationships between cp-*ndh* deletions and *ndh* genes that were transferred to the mt genome

**Figure 5:**
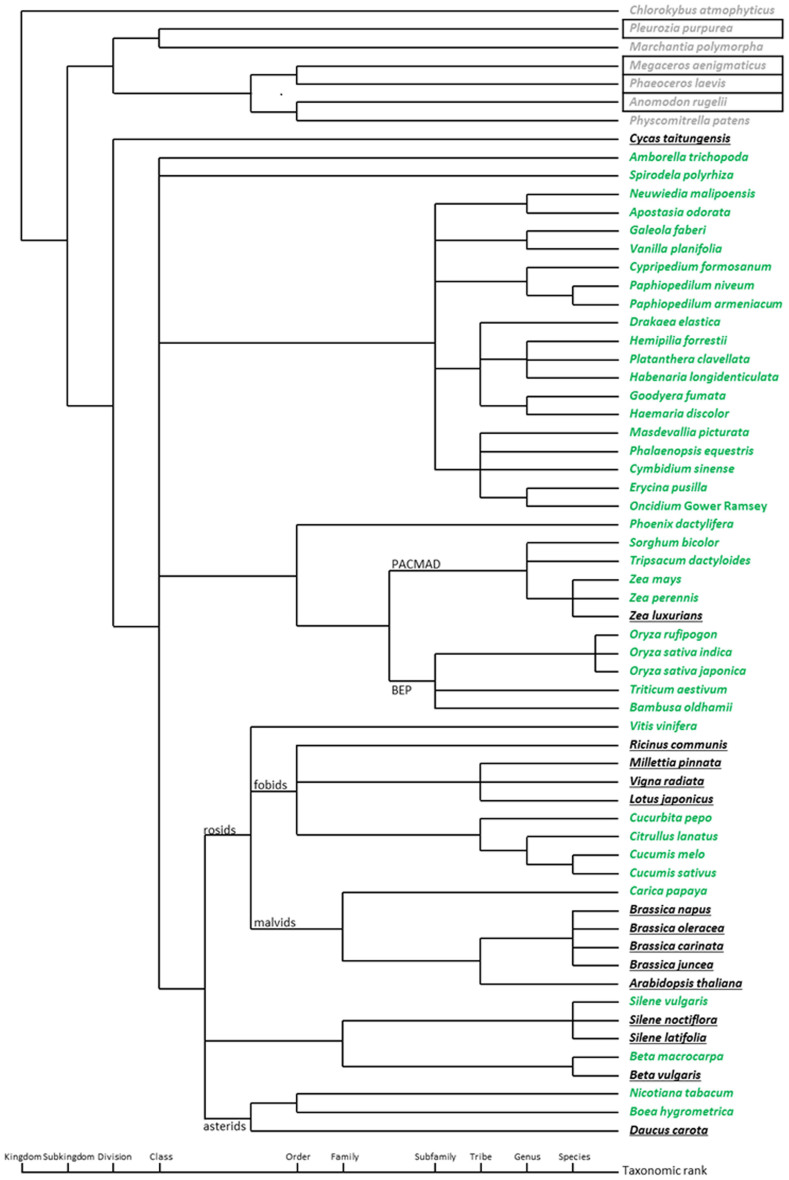
Transfer of chloroplast DNA, including *ndh* genes, to mt genomes within Kingdom Plantae. The published mitochondrial and chloroplast genomes were downloaded from NCBI. The species names in the boxes indicate that the cp genome is unavailable. The cp and mt genomes were compared using BLASTN. The names in gray indicate that no cp DNA sequences were found in the mt genome. The names that are underlined in black indicate that cp DNA was found in the mt genome but that the *ndh* genes were not. The names in green indicate the presence of *ndh* genes in the mt DNA.

An evolutionary snapshot of *ndh* gene transfer between the cp and mt genomes was taken by comparing the published mt genomes and the available cp genomes. All of the Tracheophyta (vascular plant) mt genomes contained partial cp genome fragments (Figure 5, species names in black and green). Twenty-two published Tracheophyta mt genomes contained some portion of *ndh* DNA. The length of the *mt-ndh* genic regions reflected the taxonomic relationships (Supplementary Table S3). For *Oryza*, the presence of *ndh* sequences in the mt genome correlated at the genus level (Supplementary Table S3). Based on a phylogenetic analysis of their cp genomes, *Bambusa oldhamii* and *Oryza sativa* were more closely related to each other than the other Poaceae species. They have a similar *ndh* transfer pattern, and the presence of *ndhJ*, *ndhK* and *ndhC* in both their mt genomes. However, the appearance of mt genome *ndh* sequences at the species level was different in the *Zea* genus (Figure 5)^32^; transferred *ndh* fragments were found within *Zea*, except *Zea luxurians*. A similar pattern was seen in *mt*-*ndhD* in *P. armeniacum* and *P. niveum* (Supplementary Figure S3). These results indicate that the mt genomes among members of the same family or genera may show different *ndh* content. This phenomenon is likely due to mtDNA rearrangements^28^. One possible alternative is that the mt genomes of *P. armeniacum* and *P. niveum* are not complete. As more mt genomes are sequenced and completed, the taxonomic relationship between the *ndh* regions and the cp genome sequences can be further studied.

Of all of these mt *ndh* genes, only *mt-ndhD* in *Vitis vinifera*, *mt-ndhJ* in *Phoenix dactylifera*, *B. oldhamii*, and *Oryza* and *mt-ndhB* in *Triticum aestivum* and *Zea* are full length (Supplementary Table S3)^32^. Although some *mt-ndh* gene fragments are in frame and their transcripts have been identified in these species, they cannot complement *ndh* function in *ndh*-deleted orchids.

### A large portion of the cp genome is found in the mt genome

**Figure 6:**
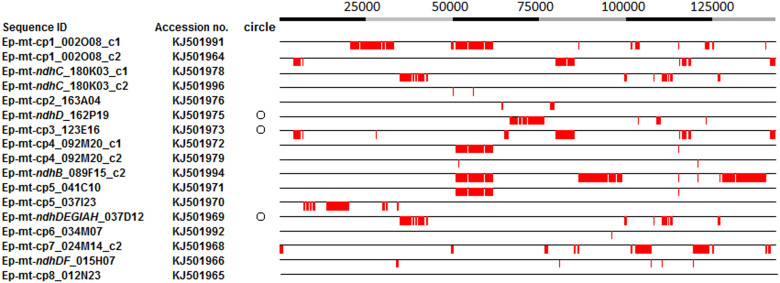
BLASTN comparison of the *Erycina pusilla* mt DNA and the *E. pusilla* cp genome. The numbers at the top are the positions of the *E. pusilla* cp genome. The red boxes indicate the aligned sequences. The numbers in the first column are the clone IDs in the *E. pusilla* BAC library. The circles indicate that the clones are circular chromosomes and have been confirmed by PCR. c1: contig 1; c2: contig 2.

The mt genomes of numerous species also contain other cp sequences in addition to the *ndh* gene fragments. Some cp-like contigs were identified among the *E. pusilla* genomic sequences and found to be similar to other plant mt genome sequences through BLASTN. Using BAC library screening, these cp-like mt DNA clones were identified in *E. pusilla* (Figure 6, Supplementary Table S4). Based on the sequences of these BAC clones, we estimate that more than 130 kb of cp genomic DNA (more that 76% of the cp genome) was transferred into the mt genome of *E. pusilla* (Figure 6). The largest cp insertion into the mt genome was 12 kb (accession no. KJ501994, containing the cp IR region).

There were 60 cp-like protein-coding genes in the *E. pusilla* mt genome BAC clones, with notable exceptions being the *matK*, *atpA*, *atpF*, *ycf3*, *rps4*, *psaI*, *petA*, and *psbJ* genes (Supplementary Table S8). According to the transcriptome results detailed above, the *ndh* DNA regions in the orchid mt genome do not encode functional proteins. Interestingly, nine cp-like mt genes (*psbI*, *rps2*, *petN*, *atpE*, *psaJ*, *psbH*, *rpl36*, *rpl22*, and *psaC*, Supplementary Table S8) contained intact gene sequences with start and stop codons but may not have required regulatory sequences.

To determine which of the cp-like mt gene fragments could be transcribed, BLASTN was used to search the *E. pusilla* transcriptome. There were 28 cp-like mt gene transcripts (Supplementary Table S8), including three intact genes: *psaJ*, *rpl36*, and *rpl22*. The *mt-psbI* sequence shared 100% identity with the cp genome sequence; therefore, we could not conclude the derivation of the transcript. The cp-like mt transcripts could be divided into two types: those containing only cp-like gene fragments and those containing mt genes (KJ501986 and KJ501987). These results indicate that these gene fragments can be transcribed but translation requires further investigation.

## Discussion

According to our results, a whole-cell transcriptome database can provide useful information for investigation of *ndh* genes in orchid genomes. However, transcriptome data should be interpreted with caution for several reasons. First, it is difficult to determine the source genome (i.e., nuclear or organellar) from transcriptome data. Second, the transcripts identified may not be full-length, may contain untranslated portions, or may not correlate with the cp genomic sequences. Third, transcriptome profiles differed between species within a subfamily. For example, in the Cypripedioideae, all 11 *ndh* genes were identified in *Cypripedium* but only 7 were identified in *Paphiopedilum*. Fourth, source materials (roots, different stages of leaves, etc.) also influence a transcription profile. Although some of the transcriptome data did not show full-length *ndh* genes, this does not necessarily mean that these genes were lost or truncated from these species. Because of these vagaries, results deduced from transcriptomes need to be confirmed by targeted sequencing of genomic DNA.

Comparison of the transcriptomes and cp genomes yielded some information; however, cp genome sequences from additional orchid species across the five subfamilies will be required to better understand the structures of *ndh* genes among orchid subfamilies. Our data also indicate that species within the same subfamily may have different *ndh* gene profiles.

According to the orchid cp genome data, cp *ndh*-deleted and *ndh*-complete species exist in the Orchidaceae family. Martin and Sabater hypothesized that the function of *ndh* genes are related to terrestrial adaptations of photosynthesis^33^. However, no significant differences were observed between the cp *ndh*-deleted and *ndh*-complete orchids in our study in terms of biogeography or growth conditions (including light and water requirements). Leitch et al.^34^ reported that orchid genome size is related to taxonomy. However, terrestrial orchids have larger genome sizes than epiphytic orchids and no significant differences were observed in our study between the *ndh*-deleted and *ndh*-complete orchids in terms of genome size^34^.

Contemporary cyanobacteria encode several thousand genes, but only 20 to 200 of these genes have been retained in modern plastid genomes^1^. A massive number of chloroplast-originating genes, including those for photosynthesis, were transferred to the nucleus after the endosymbiosis of the cyanobacterial ancestor^1^,^35^. According to our results, cp *ndh* gene fragments exist in the mt genome of cp *ndh*-deleted orchids that are not translated to functional proteins. One possible reason for this phenomenon could be that these cp *ndh* genes were transferred to the nucleus. This phenomenon has been observed in other cases of cp gene deletion. The cp *rpl22* genes have been lost in some rosid species^36^; however, the nuclear *rpl22* gene of these rosid species remains functional. This gene can be transcribed and translated into a protein that is targeted to the chloroplast, where it functions in protein synthesis^36^. Three distinct pathways for loss of cp genes have been studies so far: a) transfer to the nucleus (*infA*, *rpl22*, *rpl32*, *rpoA*), b) substitution of a nuclear encoded mitochondrial targeted gene (*rps16*) and c) substitution of a nuclear gene for a plastid gene (*accD*, *rpl23*)^36^. Therefore, we have investigated these different possibilities and transfer of *ndh* genes to the mitochondrial genome.

Another possibility is that there are no *ndh* genes in some species of orchid. No complete and functional *ndh* transcripts have been identified in the *ndh*-deleted orchid transcriptomes^21^,^37^,^38^. Although *ndh* proteins play an important role in cyclic electron transport in tobacco and *M. polymorpha*, there are no significant growth differences between the wild type and *ndh*-deleted transformants^6^,^39^,^40^,^41^. This is because there is an alternative PSI cyclic electron transport pathway: the proton gradient regulation 5 (PGR5)/PGR5-like photosynthetic phenotype 1 (PGRL1)-dependent antimycin A-sensitive pathway^7^,^42^,^43^. This pathway is partly redundant with the NDH complex-dependent pathway^6^. Therefore, the *ndh*-deleted transformants may be able to use this pathway for cyclic electron transport.

Not all of the *ndh* genes were found in the transcriptome, likely because these genes are expressed at low levels and/or only during specific developmental stages or under specific growth conditions. Genome sequences may be more useful because they represent complete genetic complements. We performed NGS sequencing of the *E. pusilla* genome and obtained 15 Gb of sequence data, which corresponds to about 10 times coverage of the genome. No nuclear *ndh* gene could be identified in the assembled NGS data. Nonetheless, because we have not completed the assembly of a high-quality draft genome, it is difficult to conclude whether any functional *ndh* genes exist in the nuclear genome. A concrete conclusion can only be made when the whole genome sequences are available for *E. pusilla*.

According to our result, most of the genes transferred to the mt genome are highly similar to those within the cp genome, indicating that these DNA sequences were transferred to the mt genome more recently than the sequences that contained more insertions/deletions and mutations. The nine proteins (psbI, rps2, petN, atpE, psaJ, psbH, rpl36, rpl22, and psaC, Supplementary Table S8) that are encoded by these genes are smaller than are the other cp genome-encoded proteins (29 to 236 a.a., average = 88 a.a.); therefore, their genetic integrity may be easier to maintain. In most Tracheophyta, small cp genome fragments have been identified in mt genomes. In rice, there are 16 cp genome segments in the mt genome with sizes ranging from 32 bp to 6.8 kb^44^. In maize, many short cp segments (17 to 187 bp) have been transferred to the mt genome^24^. In palm, there are more than 100 fragments of chloroplast origin ranging in size from 50 bp to 6 kb^45^. This large amount of synteny is one of the reasons that the *ndh*-like pseudogenes could be identified. The transfer of cp DNA to mt DNA is widespread in seed plants^45^,^46^,^47^,^48^,^49^,^50^,^51^,^52^,^53^. Based on currently published mt genomes, the *Amborella* mt genome has the highest amount of cp DNA of all of the *ndh* genes (138 kb)^48^. Two Cucurbitaceae mt genomes contain long cp genome transfers: *C. pepo* (>113 kb, five *ndh* genes)^49^ and *C. sativus* (71 kb, four *ndh* genes)^46^. In the palm *P. dactylifera* mt genome, there are 74 kb of cp genomic DNA, which encompass six *ndh* pseudogenes^45^.

More than 130 kb of cp genomic DNA has been transferred into the mt genome in *E. pusilla.* A previous study indicated that larger mt genomes contain more cp DNA^46^. We propose that the size and structure of the *E. pusilla* mt genome may be large and complicated. A complete sequence of the mt genome of *E. pusilla* will increase our understanding of this phenomenon. In the near future, NGS sequencing of the BAC library will continue to facilitate investigations of the genomic complexity in *E. pusilla*.

Most cp-like mt genes cannot translate functional proteins. In the *Amborella* mt genome, which contains 197 foreign protein-coding genes, only 50 (25%) are full-length and contain open reading frames^48^. These 50 genes are predominantly short, suggesting that many remain intact by chance. The function of cp-like mt DNA sequences include tRNA-encoding genes^50^,^51^, the promoter of *NADH dehydrogenase subunit 9* (*nad9*)^52^ and new chimeric mitochondrial genes that were generated by recombination^53^. It is still unknown why large cp DNA fragments (>1 kb) are transferred to mt DNA or why this phenomenon is frequent in orchids. Although some of the transferred tRNA genes are functional in mitochondria^50^,^51^, fates of protein coding genes require further investigations.

Previous studies have indicated that autotrophic Epidendroideae orchid cp genomes no longer encode all of the *ndh* genes. Using current orchid transcriptome databases and cp genomic sequences, we demonstrated that the complete *ndh* gene fragment set still exists in some orchid family members. During evolution, several orchids experienced *ndh* gene deletions, but these deletions are not correlated with orchid taxonomy. Based on the sequences and gene structures of the *ndh* genes and gene fragments that remain in the cp genomes, we conclude that these deletion events were independent. Because cyclic electron transport pathway is redundant with the NDH complex-dependent pathway, there is no evolutionary pressure to maintain functional copies of *ndh* genes in other genomes in sharp contrast to essential genes required for chloroplast protein synthesis that were transferred from chloroplast to the nuclear genome. In all of the sequenced orchid genomes, the *ndh* gene fragments were transferred to the mitochondrial genomes. These transfers were not directly linked to the *ndh* deletions in the cp genome. The *ndh* genes that were transferred to the mt genome have been mutated and truncated and do not complement the *ndh* gene function. Furthermore, the *E. pusilla* orchid mt genome contains the largest number of cp genome sequences among the published genomes. This result suggests that in *E. pusilla*, cp-to-mt gene fragment transfers are more frequent. Together, these findings indicate that the mt genomes of orchids (or at the very least, of *E. pusilla*) are unique and warrant further investigations.

## Methods

### Identification of 16 orchid *ndh* transcripts from public orchid transcriptome databases

To understand the *ndh* genes in the current public orchid transcriptome database, a well-studied monocot species, banana (*Musa acuminata*), was used to blast target sequences. Eleven ndh amino acid sequences were used as templates for TBLASTN searches (Accession no. HF677508)^54^. These amino acid sequences were used to identify the putative *ndh* gene transcripts from public transcriptome databases^21^,^37^,^38^. The cDNA sequences that were identified in this study were targeted from BLAST searches. The sequence ID is listed in Supplementary Table S2.

### Plant and BAC DNA preparation

*V. planifolia*, *H. longidenticulata*, *G. fumata*, *P. armeniacum*, *P. niveum*, *C. formosanum*, *M. picturata*, and *E. pusilla* leaves were used as the material for DNA extraction *via* the cetyltrimethyl ammonium bromide method^55^. The BAC plasmids for Illumina sequencing were isolated using the NucleoBond BAC 100 Kit (NucleoSpin Blood, Macherey-Nagel, Düren, Germany).

### High-throughput sequencing

The above orchid genomic and BAC DNA fragments were sequenced by an Illumina high-throughput sequencing platform (Illumina, San Diego, CA, USA). The paired-end sequencing libraries were constructed by a Nextera XT DNA Sample Preparation Kit according to the instruction manual (Illumina). Briefly, 10 μg of purified DNA was fragmented using the Amplicon Augment Mix (Illumina). Fragmentation reactions were achieved by incubation at 55°C for 5 min, followed by neutralization in buffer at room temperature for 5 min. The neutralized DNA fragments were amplified *via* a limited-cycle PCR program involving 12 cycles of denaturation at 95°C for 10 s, primer annealing at 55°C for 30 s, and extension at 72°C for 30 s, during which the index primers that were required for cluster formation were also added. The amplified DNA was purified using AMPure XP beads (Beckman Coulter, Indianapolis, IN, USA). The fragment sizes and concentrations of the libraries were determined by a 2100 Bioanalyzer with a High Sensitivity DNA Assay Kit (Agilent Technologies, Santa Clara, CA, USA) and quantitative PCR (Applied Biosystems, Carlsbad, CA, USA). Subsequently, the libraries with insert sizes of 500 to 600 bp were denatured with NaOH and sequenced at read lengths of 250 bases from both ends using a MiSeq Personal Sequencer (Illumina).

Because the mean insert size of each library was approximately 500 to 600 bp, the ends of many reads (250 bp*2) overlapped due to the variability in the insert size. Therefore, the cleaned reads were classified into two groups: overlapping paired-end reads and non-overlapping paired-end reads. The overlapping paired-end reads were further merged into long reads (400 to 500 bp) by FLASH (version 1.2.7) before assembly^56^. The remaining paired-end reads were used to link the contigs into scaffolds. Newbler (version 2.9) was used to assemble the long reads along with the non-overlapping paired-end reads into contigs and scaffolds. Detailed information on the high-throughput sequencing is provided in Supplementary Table S5. The assembly sequences from the genomic DNA of seven orchids are defined as whole genome sequences of seven orchids for the further analysis.

### Complete chloroplast genomes of seven orchids

The cp DNA contigs were identified using the *E. pusilla* cp genome sequence to screen the whole genome sequences of seven orchids by BLASTN^19^. Those contigs with a high coverage were used as the skeleton of the cp genomes. The gaps between the contigs were filled by sequences that were derived from PCR. The cp genome was annotated using the Dual Organellar GenoMe Annotator (DOGMA)^57^. For genes with a low sequence identity, manual annotation was performed after identifying the positions of the start and stop codons as well as the translated amino acid sequence using the chloroplast/bacterial genetic code. The annotated genome sequences were submitted to NCBI. The accession numbers for the assembled chloroplast genomes are listed in Supplementary Table S4.

### Identification of orchid non-cp *ndh* genes

Because no whole nuclear genome sequence of an orchid has been published, regions containing *ndh-*coding sequences in the genomes of the nucleus and other organelles (non-cp *ndh* genes) have not been identified. To identify the non-cp *ndh* genes, 11 banana (*M. acuminata*) ndh amino acid sequences were used as templates for TBLASTN searches of the orchid genomic sequences (Accession no. HF677508)^54^. The identified contigs were analyzed by BLASTN searches with the sequences of their corresponding cp genomes to confirm that these sequences were not cp genomic sequences. The mt DNA sequences were determined by comparisons of the regions flanking the *ndh* gene fragments with the mt genome sequences in the NCBI database using BLASTN. In addition, the raw read count was used to distinguish the source of the DNA using the correlation between the number of high-throughput sequencing reads per sequence and the source (multiple copies of organelles vs. single genome, Supplementary Table S5) of the DNA when total DNA was used to exclude the cp genome sequence^58^. The remaining candidate contigs were confirmed by NCBI BLASTN to ensure the sequences were mt DNA.

The non-cp *ndh* sequences which derived from genomic sequencing in *E. pusilla* were used to design the specific primers. These primers were used to screen the BAC library. Using this strategy, there was no need to isolate the nuclei or mitochondria or to purify the genomic DNA without cp genome contamination. The sequences were annotated by BLASTX using the *Arabidopsis* protein database. The figures of the 446 annotated and aligned DNA positions were drawn using PowerPoint (Microsoft, 14 447 Redmond, WA). The flow chart is shown in Supplementary Figure S5.

### Phylogenetic analysis

To investigate the relationships between the orchids in this report, the combined chloroplast *rbcL*, *matK*, *psaA*, and *psaB* nucleotide sequences were used for the phylogenetic analysis. The consensus sequences were aligned using ClustalW2 (http://www.clustal.org/) and concatenated. Subsequently, the maximum-likelihood phylogeny was estimated using a web version of PhyML 3.0 (http://www.atgc-montpellier.fr/phyml/) using the multiple sequence alignments^59^. The General Time Reversible model was used as a substitution model to build the phylogenetic tree. The bootstrap values were obtained from 1000 bootstrap replicates and are presented as percentages. Finally, the phylogenetic tree was plotted by TreeVector (http://supfam.cs.bris.ac.uk/TreeVector/).

### Identification of the *ndh* genes and cp-like DNA from the published plant mt genomes

To identify the *ndh* genes in the published plant mt genomes, the 43 complete mt genomes and the cp genomes of these species or related species in the same genus were downloaded from the NCBI database. The species and accession nos. are listed in Supplementary Table S9. The cp and mt genomes were compared using BLASTN to identify the cp DNA in the mt genomes.

### Identification of the BAC clones that contain the *ndh* genes and cp-like DNA in their mitochondrial DNA

The specific primers were designed to identify the BAC clones carrying the *ndh* genic regions and cp-like DNA (Supplementary Table S10). The BAC clones containing the *ndh* regions and cp-like DNA of interest were obtained by PCR screening from the super pool, plate, row and spot^31^. These primers were also used to screen the *O.* ‘Gower Ramsey' BAC library to identify *ndh* clones.

## Author Contributions

M.C.S., M.T.C., H.D. and C.S.L. designed the research. J.J.W.C., Y.T.H., S.Y.L., M.C.S. and M.T.C. performed next generation sequencing. J.J.W.C., Y.T.H., S.Y.L., M.C.S., M.T.C., C.F.L. and C.Y.C. performed bioinformatics analysis. H.D., W.J.C., C.T.H., D.C.L., F.H.W. and M.L.C. performed chloroplast genome annotation and uploaded to NCBI. W.J.C., C.T.H., D.C.L. and F.H.W. performed BAC library screening and sequencing. G.K.S.W. and M.K.D. established the 1000 plants transcriptome database. M.C.S., W.J.C., H.D., M.K.D., V.A.A. and C.S.L. wrote the paper.

## Supplementary Material

Supplementary Information Supplementary Information
