# Supplementary material for: The location and translocation of ndh genes of chloroplast origin in the Orchidaceae family
Source: Sci Rep. 2015 Mar 12;5:9040. doi: 10.1038/srep09040 (PMC4356964; doi:10.1038/srep09040)

Supplementary Information

The location and translocation of *ndh* genes of chloroplast origin in the Orchidaceae family

Choun-Sea Lin[1,§,*], Jeremy J.W. Chen[2,*], Yao-Ting Huang[3,*], Ming-Tsair Chan[1,4,*], Henry Daniell[5,*], Wan-Jung Chang[1,*], Chen-Tran Hsu[1], De-Chih Liao[1], Fu-Huei Wu[1], Sheng-Yi Lin[2], Chen-Fu Liao[3], Michael K. Deyholos[6], Gane Ka-Shu Wong[6,7,8], Victor A. Albert[9], Ming-Lun Chou[10], Chun-Yi Chen[1], and Ming-Che Shih[1,§]

[1]Agricultural Biotechnology Research Center, Academia Sinica, Taipei, Taiwan

[2]Institute of Biomedical Sciences, National Chung-Hsing University, Taichung, Taiwan

[3]Department of Computer Science and Information Engineering, National Chung Cheng University, Chiayi, Taiwan

[4]Academia Sinica Biotechnology Center in Southern Taiwan, Tainan, Taiwan

[5]Departments of Biochemistry and Pathology, University of Pennsylvania School of Dental Medicine, Philadelphia, PA, USA

[6]Department of Biological Sciences, University of Alberta, Edmonton, AB, Canada

[7]Department of Medicine, University of Alberta, Edmonton AB, Canada

[8]BGI-Shenzhen, Beishan Industrial Zone, Yantian District, Shenzhen, China

[9]Department of Biological Sciences, University at Buffalo, Buffalo, NY, USA

[10]Department of Life Sciences, Tzu Chi University, Hualien, Taiwan

*These authors contributed equally to this work

[§]Corresponding author

MCS: mcshih@gate.sinica.edu.tw

CSL: cslin99@gate.sinica.edu.tw

Table of Contents

Supplementary Table S1. Percentage of each *ndh* gene remaining within the transcriptomes of 16 orchid species. Page 3

Supplementary Table S2. The orchid *ndh* sequence IDs in transcriptome databases. Page 4

Supplementay Table S3. Plant cp *ndh* genes and their transfer to plant mt genomes. Page 9

Supplementary Table S4. The accession numbers of the sequences that were identified in this report. Page 10

Supplementary Table S5. Sequencing coverage of the seven assembled chloroplast genomes. Page 12

Supplementary Table S6. Summary of the transcriptome, chloroplast and mitochondrial genome sequences of *Vanilla planifolia* (*ndh*-deleted), *Paphiopedilum armeniacum* (*ndh*-deleted), *Cypripedium formosanum* (*ndh*-complete), *Habenaria longidenticulata*, *Goodyera fumata*, *Erycina pusilla* (*ndh*-deleted) and *Masdevallia picturata* (*ndh*-complete). Page 13

Supplemental Table S7. Mitochondrial-derived *ndh* transcripts. Page 14

Supplementary Table S8. Summary of 68 cp-like mt gene fragments. Page 15

Supplementary Table S9. Mitochondria and chloroplast genome accession numbers in identifying the *ndh* genes and cp-like DNA from the published plant mt genomes. Page 17

Supplementary Table S10. Primer list for BAC clone screening. Page 18

Supplemental Figure S1. Gene maps of orchid chloroplast genomes. Page 19

Supplementary Figure S2. The cp *ndhJ-K-C* genes were transferred to the plant mt genome. Page 22

Supplementary Figure S3. The cp *ndhF-D-E-G-I-A-H* region was transferred to the plant mt genome. Page 23

Supplementary Figure S4. The cp *ndhB* gene fragment was transferred to the plant mt genome. Page 24

Supplementary Figure S5. Flowchart of sequence annotation. Page 25

Supplementary Table S1. Percentage of each *ndh* gene remaining within the transcriptomes of 16 orchid species.[1]

ndh gene*	F	D	E	G	I	A	H	J	K	C	B
**Apostasioideae**											
Apostasia shenzhenica	-	96	-	-	80	40	-	64	86	100	34
Neuwiedia malipoensis	62	100	84	40	83	78	83	31	-	100	96
**Vanilloideae**											
Vanilla shenzhenica	-	-	-	-	-	-	-	-	35	-	8
Galeola faberi	-	-	-	-	-	16	-	-	-	40	6
Vanilla planifolia	-	-	-	-	-	-	-	25	15	44	8
**Cypripedioideae**											
Cypripedium singchii	7	94	100	94	83	8	74	98	98	100	65
Paphiopedilum armeniacum	-	30	-	-	-	6	80	100	100	100	62
**Orchidoideae**											
Hemipilia forrestii	58	71	64	46	100	76	78	-	4	-	44
Habenaria delavayi	50	84	100	100	100	88	58	-	89	100	96
Drakaea elastica	-	20	-	-	-	-	-	-	76	-	22
Goodyera pubescens	50	80	100	100	94	91	100	100	91	100	16
Platanthera clavellata	25	87	10	82	94	92	100	98	91	100	64
**Epidendroideae**											
Masdevallia yuangensis	67	79	82	72	68	44	88	100	100	100	100
Phalaenopsis equestris	10	30	-	24	20	-	-	96	26	58	74
Cymbidium sinense	79	100	42	84	100	90	100	100	94	91	94
Erycina pusilla	-	22	-	44	-	-	-	47	-	91	26

[1]No. of aligned amino acids/no. of total amino acids. The sequence IDs are shown in Supplementary Table S2.

*The percentage amino acid identity is in comparison with the corresponding *ndh* gene that was translated from banana.

'-'indicates genes that were not found in the transcriptome of a species.

Supplementary Table S2. The orchid ndh sequence IDs in transcriptome databases.

Species	*ndh* transcripts			Material information	Reference
	ndhJ-K-C region	ndhF-D-E-G-I-A-H region	ndhB		
**Apostasioideae**					
Apostasia shenzhenica	>Unigene69269_Ap_fb	>Unigene41918_Ap_fb	>Unigene29767_Ap_fb	mature flower buds	Tsai et al. 2013
	>Unigene87795_Ap_fb	>Unigene53823_Ap_fb	>Unigene28875_Ap_fb		
	>Unigene88477_Ap_fb	>Unigene72742_Ap_fb	>Unigene79060_Ap_fb		
	>Unigene23503_Ap_fb	>Unigene73556_Ap_fb	>Unigene29163_Ap_fb		
	>Unigene11539_Ap_fb	>Unigene76972_Ap_fb	>Unigene2188_Ap_fb		
	>Unigene90084_Ap_fb	>Unigene79556_Ap_fb	>Unigene9034_Ap_fb		
	>Unigene46738_Ap_fb	>Unigene81057_Ap_fb	>Unigene3335_Ap_fb		
	>Unigene28081_Ap_fb	>Unigene85689_Ap_fb			
	>Unigene52144_Ap_fb				
Neuwiedia malipoensis	>Unigene117027_Ne_fb	>Unigene116626_Ne_fb	>Unigene156751_Ne_fb	mature flower buds	Tsai et al. 2013
	>Unigene116386_Ne_fb	>Unigene120297_Ne_fb	>Unigene13393_Ne_fb		
	>Unigene80802_Ne_fb	>Unigene129251_Ne_fb	>Unigene28019_Ne_fb		
	>Unigene92339_Ne_fb	>Unigene13695_Ne_fb	>Unigene140983_Ne_fb		
		>Unigene139703_Ne_fb	>Unigene156912_Ne_fb		
		>Unigene140737_Ne_fb	>Unigene105627_Ne_fb		
		>Unigene143156_Ne_fb	>Unigene43751_Ne_fb		
		>Unigene146834_Ne_fb	>Unigene7472_Ne_fb		
		>Unigene148454_Ne_fb			
		>Unigene154307_Ne_fb			
		>Unigene157226_Ne_fb			
		>Unigene48454_Ne_fb			
		>Unigene65423_Ne_fb			
		>Unigene65942_Ne_fb			
		>Unigene66460_Ne_fb			
		>Unigene72149_Ne_fb			
		>Unigene73733_Ne_fb			
		>Unigene80484_Ne_fb			
		>Unigene84449_Ne_fb			
		>Unigene85091_Ne_fb			
		>Unigene86866_Ne_fb			
		>Unigene94366_Ne_fb			
		>Unigene96329_Ne_fb			
		>Unigene99485_Ne_fb			

Species	*ndh* transcripts			Material information	Reference
	ndhJ-K-C region	ndhF-D-E-G-I-A-H region	ndhB		
Cypripedioideae					
Cypripedium singchii	>Unigene123012_Ch_fb<br>>Unigene126895_Ch_fb<br>>Unigene91373_Ch_fb<br>>Unigene124112_Ch_fb<br>>Unigene44022_Ch_fb<br>>Unigene54875_Ch_fb<br>>Unigene79119_Ch_fb<br>>Unigene64311_Ch_fb	>Unigene108704_Ch_fb<br>>Unigene86389_Ch_fb<br>>Unigene38615_Ch_fb<br>>Unigene132771_Ch_fb<br>>Unigene117910_Ch_fb<br>>Unigene110223_Ch_fb<br>>Unigene133375_Ch_fb<br>>Unigene117089_Ch_fb<br>>Unigene15981_Ch_fb<br>>Unigene120965_Ch_fb<br>>Unigene76575_Ch_fb<br>>Unigene117252_Ch_fb<br>>Unigene36780_Ch_fb<br>>Unigene50561_Ch_fb<br>>Unigene35987_Ch_fb	>Unigene81774_Ch_fb<br>>Unigene116499_Ch_fb<br>>Unigene76103_Ch_fb<br>>Unigene120814_Ch_fb<br>>Unigene78391_Ch_fb<br>>Unigene121760_Ch_fb	mature flower buds	Tsai et al. 2013
Paphiopedilum armeniacum	>Unigene17537_Pa_fb<br>>Unigene19265_Pa_fb	>Unigene117683_Pa_fb<br>>Unigene86259_Pa_fb<br>>Unigene105103_Pa_fb<br>>Unigene102973_Pa_fb<br>>Unigene90098_Pa_fb	>Unigene105265_Pa_fb<br>>Unigene66033_Pa_fb<br>>Unigene102147_Pa_fb<br>>Unigene19856_Pa_fb<br>>Unigene102907_Pa_fb<br>>Unigene42239_Pa_fb<br>>Unigene57666_Pa_fb<br>>Unigene36063_Pa_fb	mature flower buds	Tsai et al. 2013

Species	*ndh* transcripts			Material information	Reference
	ndhJ-K-C region	ndhF-D-E-G-I-A-H region	ndhB		
Vanilloideae					
Vanilla planifolia	>scaffold-THDM-2043406-Vanilla_planifolia<br>>scaffold-THDM-2006247-Vanilla_planifolia		>scaffold-THDM-2032069-Vanilla_planifolia	No material information	Johnson et al. 2012
Vanilla shenzhenica	>Unigene147494_Va_fb<br>>Unigene123_Va_fb<br>>Unigene23174_Va_fb<br>>Unigene81512_Va_fb		>Unigene54646_Va_fb	mature flower buds	Tsai et al. 2013
Galeola faberi	>Unigene71422_Ga_fb<br>>Unigene107437_Ga_fb<br>>Unigene20405_Ga_fb	>Unigene57147_Ga_fb<br>>Unigene27278_Ga_fb<br>>Unigene50721_Ga_fb		mature flower buds	Tsai et al. 2013

Species	*ndh* transcripts			Material information	Reference
	ndhJ-K-C region	ndhF-D-E-G-I-A-H region	ndhB		
**Orchidoideae**					
Drakaea elastica	>scaffold-XZME-2073613-Drakaea_elastica	>scaffold-XZME-2013708-Drakaea_elastica	>scaffold-XZME-2067623-Drakaea_elastica	leaves, flowers, and buds	Johnson et al. 2012
Goodyera pubescens	>scaffold-VTUS-2052198-Goodyera_pubescens	>scaffold-VTUS-2008714-Goodyera_pubescens	>scaffold-VTUS-2027247-Goodyera_pubescen	young leaves	Johnson et al. 2012
		>scaffold-VTUS-2049624-Goodyera_pubescens	>scaffold-VTUS-2017751-Goodyera_pubescens		
		>scaffold-VTUS-2050414-Goodyera_pubescens	>scaffold-VTUS-2024711-Goodyera_pubescens		
		>scaffold-VTUS-2044337-Goodyera_pubescens	>scaffold-VTUS-2026918-Goodyera_pubescens		
		>scaffold-VTUS-2008713-Goodyera_pubescens	>scaffold-VTUS-2040139-Goodyera_pubescens		
		>scaffold-VTUS-2043164-Goodyera_pubescens	>scaffold-VTUS-2010245-Goodyera_pubescens		
		>scaffold-VTUS-2037255-Goodyera_pubescens	>scaffold-VTUS-2008415-Goodyera_pubescens		
Habenaria delavayi	>Unigene11883_Ha_fb	>Unigene103331_Ha_fb	>Unigene100905_Ha_fb	mature flower buds	Tsai et al. 2013
	>Unigene2826_Ha_fb	>Unigene104496_Ha_fb	>Unigene11008_Ha_fb		
	>Unigene84798_Ha_fb	>Unigene105393_Ha_fb	>Unigene12605_Ha_fb		
		>Unigene109799_Ha_fb	>Unigene1993_Ha_fb		
		>Unigene29980_Ha_fb	>Unigene2468_Ha_fb		
		>Unigene30495_Ha_fb	>Unigene45846_Ha_fb		
		>Unigene38599_Ha_fb	>Unigene67111_Ha_fb		
		>Unigene40484_Ha_fb			
		>Unigene40484_Ha_fb			
		>Unigene50000_Ha_fb			
		>Unigene63124_Ha_fb			
		>Unigene83484_Ha_fb			
		>Unigene83500_Ha_fb			
		>Unigene8483_Ha_fb			
		>Unigene93473_Ha_fb			
		>Unigene97680_Ha_fb			
		>Unigene99504_Ha_fb			
Hemipilia forrestii	>Unigene107848_Hem	>Unigene100534_Hem	>Unigene104501_Hem	mature flower buds	Tsai et al. 2013
	>Unigene95563_Hem	>Unigene104197_Hem	>Unigene64030_Hem		
	>Unigene108373_Hem	>Unigene1054_Hem	>Unigene74470_Hem		
	>Unigene24273_Hem	>Unigene107907_Hem	>Unigene8168_Hem		
	>Unigene49469_Hem	>Unigene110892_Hem	>Unigene86982_Hem		
		>Unigene17478_Hem	>Unigene89656_Hem		
		>Unigene24809_Hem			
		>Unigene29088_Hem			
		>Unigene36004_Hem			
		>Unigene37397_Hem			
		>Unigene39152_Hem			
		>Unigene39228_Hem			
		>Unigene46424_Hem			
		>Unigene53948_Hem			
		>Unigene63135_Hem			
		>Unigene6863_Hem			
		>Unigene74818_Hem			
		>Unigene7947_Hem			
		>Unigene81760_Hem			
		>Unigene82799_Hem			
		>Unigene87191_Hem			

		>Unigene91361_Hem			
		>Unigene99622_Hem			
Platanthera clavellata	>scaffold-MTHW-2006569-Platanthera_clavella	>scaffold-MTHW-2054067-Platanthera_clavellata	>scaffold-MTHW-2005097-Platanthera_clavell	young leaves	Johnson et al. 2012
		>scaffold-MTHW-2012569-Platanthera_clavellata	>scaffold-MTHW-2005096-Platanthera_clavellata		
		>scaffold-MTHW-2012285-Platanthera_clavellata	>scaffold-MTHW-2033930-Platanthera_clavellata		
		>scaffold-MTHW-2002299-Platanthera_clavellata	>scaffold-MTHW-2030317-Platanthera_clavellata		
		>scaffold-MTHW-2002298-Platanthera_clavellata	>scaffold-MTHW-2002472-Platanthera_clavellata		
		>scaffold-MTHW-2044147-Platanthera_clavellata	>scaffold-MTHW-2049556-Platanthera_clavellata		
		>scaffold-MTHW-2010146-Platanthera_clavellata_r	>scaffold-MTHW-2011571-Platanthera_clavellata		
		>scaffold-MTHW-2039772-Platanthera_clavellata			
		>scaffold-MTHW-2048797-Platanthera_clavellata			
		>scaffold-MTHW-2039314-Platanthera_clavellata			
		>scaffold-MTHW-2038829-Platanthera_clavellata			
		>scaffold-MTHW-2021258-Platanthera_clavellata			

Species	*ndh* transcripts			Material information	Reference
	ndhJ-K-C region	ndhF-D-E-G-I-A-H region	ndhB		
Epidendroideae					
Cymbidium sinense	>Unigene91693_Cym_fb	>Unigene97292_Cym_fb	>Unigene102171_Cym_fb	mature flower buds	Tsai et al. 2013
	>Unigene58080_Cym_fb	>Unigene95349_Cym_fb	>Unigene108654_Cym_fb		
	>Unigene91202_Cym_fb	>Unigene104107_Cym_fb			
		>Unigene53349_Cym_fb			
		>Unigene38535_Cym_fb			
		>Unigene96064_Cym_fb			
		>Unigene82889_Cym_fb			
		>Unigene78331_Cym_fb			
		>Unigene57645_Cym_fb			
		>Unigene98811_Cym_fb			
		>Unigene477_Cym_fb			
		>Unigene108035_Cym_fb			
Erycina pusilla	>Unigene2294_All	>Unigene48484_All	>Unigene56611_All	Root, leaves, flowers, fruits	Chou et al. 2013
	>Unigene18133_All	>Unigene66487_All	>Unigene63750_All		
	>Unigene18740_All				
	>Unigene53575_All				
Masdevallia yuangensis	>scaffold-JSAG-2016102-Masdevallia_yuangen	>scaffold-JSAG-2080352-Masdevallia_yuangensis	>scaffold-JSAG-2017126-Masdevallia_yuange	buds, leaves, roots	Johnson et al. 2012
		>scaffold-JSAG-2015140-Masdevallia_yuangensis	>scaffold-JSAG-2017125-Masdevallia_yuangensis		
		>scaffold-JSAG-2079074-Masdevallia_yuangensis			
		>scaffold-JSAG-2078946-Masdevallia_yuangensis			
		>scaffold-JSAG-2063713-Masdevallia_yuangensis			
		>scaffold-JSAG-2073565-Masdevallia_yuangensis			
		>scaffold-JSAG-2065747-Masdevallia_yuangensis			
		>scaffold-JSAG-2014459-Masdevallia_yuangensis			
		>scaffold-JSAG-2071180-Masdevallia_yuangensis			
		>scaffold-JSAG-2071180-Masdevallia_yuangensis			
Phalaenopsis equestris	>Unigene80637_Pe_fb	>Unigene70210_Pe_fb	>Unigene84064_Pe_fb	mature flower buds	Tsai et al. 2013
	>Unigene76415_Pe_fb	>Unigene55346_Pe_fb	>Unigene93337_Pe_fb		
		>Unigene82158_Pe_fb	>Unigene11847_Pe_fb		
		>Unigene7851_Pe_fb			
		>Unigene91525_Pe_fb			

Supplementary Table S3. Plant cp *ndh* genes and their transfer to plant mt genomes.

Species	*ndh* genes										
	F	***D***	***E***	***G***	***I***	***A***	***H***	***J***	***K***	***C***	***B***
**Chloroplast genome**											
**Vanilloideae**											
Vanilla planifolia	-	-	-	-	-	-	-	-	-	-	16
**Cypripedioideae**											
Paphiopedilum armeniacum	-	78	-	-	-	-	-	100	100	100	100
Paphiopedilum niveum	-	78	-	-	-	-	-	100	100	100	100
Cypripedium formosanum	100	100	100	100	100	100	100	100	100	100	100
**Orchidoideae**											
Habenaria longidenticulata	100	100	100	100	100	100	100	100	100	100	100
Goodyera fumata	100	100	100	100	100	100	100	100	100	100	100
**Epidendroideae**											
Erycina pusilla	-	60	-	52	-	-	32	42	-	68	43
Oncidium 'Gower Ramsey'	-	65	99	100	48	40	15	43	13	100	99
Phalaenopsis equestris	-	50	-	24	18	-	-	98	27	60	79
*Cymbidium sinense**	81	100	100	100	100	92	100	100	100	100	100
Masdevallia picturata	100	100	100	100	100	100	100	100	100	100	100
**Mitochondrial genome**											
**Vanilloideae**											
Vanilla planifolia	-	-	58	-	-	-	-	79	43	70	-
**Cypripedioideae**											
Paphiopedilum armeniacum	-	82	-	-	-	49	98	100	-	100	85
Paphiopedilum niveum	50	80	50	-	-	-	79	98	91	99	100
Cypripedium formosanum	-	-	-	-	-	-	-	-	-	63	-
**Orchidoideae**											
Habenaria longidenticulata	59	64	-	-	-	36	-	-	-	-	-
Goodyera fumata	-	35	100	-	99	44	8	96	-	80	7
**Epidendroideae**											
Masdevallia picturata	75	98	100	100	100	94	99	-	-	30	82
Erycina pusilla	41	78	100	98	28	91	100	48	-	96	50
Oncidium 'Gower Ramsey'	70	30	-	-	-	-	-	-	-	-	97
**Others**											
Spirodela polyrhiza	-	-	-	-	-	-	-	-	49	58	-
Phoenix dactylifera	-	-	-	-	100	44	-	100	96	-	-
Bambusa oldhamii	-	-	-	-	-	-	-	100	87	48	-
Oryza rufipogon	-	-	-	-	-	-	-	100	95	34	-
Oryza sativa indica	-	-	-	-	-	-	-	100	95	34	-
Oryza sativa japonica	-	-	-	-	-	-	-	100	95	34	-
Triticum aestivum	-	-	-	-	-	26	-	-	-	-	100
Sorghum bicolor	-	-	-	-	33	-	-	-	88	98	-
Tripsacum dactyloides	-	-	-	-	-	-	-	-	20	-	10
Zea mays	-	-	-	-	-	-	-	-	20	-	100
Zea perennis	-	-	-	-	-	-	-	-	20	-	100
Amborella trichopoda	87	97	98	99	100	49	100	99	99	100	100
Vitis vinifera	91	100	99	-	-	-	42	-	-	-	16
Cucurbita pepo	36	80	34	61	-	-	47	-	85	64	93
Citrullus lanatus	-	-	-	-	-	-	-	99	98	100	-
Cucumis melo	-	-	36	-	49	22	-	-	-	-	-
Cucumis sativus	-	91	59	85	11	-	-	58	-	-	100
Beta macrocarpa	-	-	-	-	-	-	-	-	83	-	-
Silene vulgaris	-	61	-	-	-	-	-	-	-	-	-
Nicotiana tabacum	-	-	-	-	-	-	12	-	-	-	85
Carica papaya	-	-	-	-	-	-	-	-	-	-	78
Boea hygrometrica	-	-	-	-	-	-	-	70	90	53	100

The light green rows of the table show the *ndh* genes in the orchid chloroplast genomes. The remainder of the table contains data from the available mitochondrial genomes. The numbers indicate the percentage of the nucleotide fragments that are similar to the *ndh* sequences.
The red numbers indicate that the *ndh* DNA is in frame and encodes the full-length *ndh* gene.
'-' indicates that there is no homolog in the mt genome.

Supplementary Table S4. The accession numbers of the sequences that were identified in this report.

Chloroplast genome Sequences

Subfamily	Name	Accession
Vanilloideae	*Vanilla planifolia*	KJ566306
Cypripedioideae	*Cypripedium formosanum*	KJ501998
	Paphiopedilum armeniacum	KJ566307
	Paphiopedilum niveum	KJ524105
Orchidoideae	*Goodyera fumata*	KJ501999
	Habenaria longidenticulata	KJ524104
Epidendroideae	*Masdevallia picturata*	KJ566305

Orchid *ndh* DNA sequences

Subfamily	Species	Name	Accession
Cypripedioideae	*Paphiopedilum niveum*	Paph_niveum_ndhB1	KJ501931
		Paph_niveum_ndhB2	KJ501932
		Paph_niveum_ndhB3	KJ501933
		Paph_niveum_ndhB4	KJ501989
		Paph_niveum_ndhB5	KJ501934
		Paph_niveum_ndhB6	KJ501935
		Paph_niveum_ndhB7	KJ501936
		Paph_niveum_ndhB8	KJ501937
		Paph_niveum_ndhE1	KJ501938
		Paph_niveum_ndhH	KJ501939
		Paph_niveum_ndhJKC2	KJ501941
		Paph_niveum_ndhJKC3	KJ501942
		Paph_niveum_ndhJ	KJ501943
	Paphiopedilum armeniacum	Paph_armeniacum_ndhJC	KJ501952
Epidendroideae	*Masdevallia picturata*	Mas_ndhF	KJ501928
		Mas_ndhJK	KJ501929

Orchid mitochondria *ndh* DNA sequences

Subfamily	Species	Name	Accession
Vanilloideae	*Vanilla planifolia*	Vp-mt-ndhJ	KJ501948
		Vp-mt-ndhK	KJ501957
		Vp-mt-ndhC	KJ501923
		Vp-mt-ndhE	KJ501959
Cypripedioideae	*Cypripedium formosanum*	Cf-mt-ndhC	KJ501922
		Cf-mt-ndhH	KJ501956
		Pa-mt-ndhCD	KJ501961
		Pa-mt-ndhAH	KJ501962
		Pa-mt-ndhB	KJ501951
		Pn-mt-ndhJKC	KJ501944
		Pn-mt-ndhD	KJ501945
Orchidoideae	*Habenaria longidenticulata*	Ha-mt-ndhA	KJ501960
		Ha-mt-ndhD	KJ501926
		Ha-mt-ndhF	KJ501954
	Goodyera fumata	Go-mt-ndhH	KJ501955
		Go-mt-ndhAIE	KJ501963
		Go-mt-ndhD	KJ501927
		Go-mt-ndhB	KJ501921
Epidendroideae	*Erycina pusilla*	Ep-mt-ndhJC	KJ501950

Epidendroideae	*Erycina pusilla*	Ep-mt-ndhC	KJ501996
		Ep-mt-ndhDEGIAH	KJ501958
		Ep-mt-ndhDF	KJ501966
		Ep-mt-ndhD	KJ501975
		Ep-mt-ndhB	KJ501994
	Goodyera fumata	Go-mt-ndhJ	KJ501949
		Go-mt-ndhC	KJ501924
	Masdevallia picturata	Ma-mt-ndhC	KJ501930
		Ma-mt-ndhHAIGED	KJ501946
		Ma-mt-ndhB	KJ501947
	Oncidium 'Gower Ramsey'	Onc-mt-ndhF	KJ501953
		Onc-mt-ndhD	KJ501925
		Onc-mt-ndhB	KJ501920

***Erycina pusilla* mitochondria BAC clone sequences**

Subfamily	Species	Name	Accession
Epidendroideae	*Erycina pusilla*	Ep-mt-cp1_002O08_c1	KJ501991
		Ep-mt-cp1_002O08_c2	KJ501964
		Ep-mt-ndhC 180K03_c1	KJ501978
		Ep-mt-ndhC 180K03_c2	KJ501996
		Ep-mt-ndhC 180K03_c3	KJ501997
		Ep-mt-cp2_163A04	KJ501976
		Ep-mt-ndhD162P19	KJ501975
		Ep-mt-cp3_123E16	KJ501973
		Ep-mt-cp4_092M20_c1	KJ501972
		Ep-mt-cp4_092M20_c2	KJ501979
		Ep-mt-ndhB 089F15_c1	KJ501993
		Ep-mt-ndhB 089F15_c2	KJ501994
		Ep-mt-ndhB 089F15_c3	KJ501995
		Ep-mt-cp5_041C10	KJ501971
		Ep-mt-cp5_037I23	KJ501970
		Ep-mt-ndhDEGIAH 037D12	KJ501969
		Ep-mt-cp6_034M07	KJ501992
		Ep-mt-cp7_024M14_c1	KJ501967
		Ep-mt-cp7_024M14_c2	KJ501968
		Ep-mt-ndhDF 015H07	KJ501966
		Ep-mt-cp8_012N23	KJ501965

***Erycina pusilla* mitochondria cDNA sequences**

Subfamily	Species	Name	Accession
Epidendroideae	*Erycina pusilla*	C211673	KJ501980
		C92325	KJ501981
		C13581	KJ501982
		C115515	KJ501983
		C117162	KJ501984
		C16074	KJ501985
		C122767	KJ501986
		C74904	KJ501987
		C167460	KJ501988
		C92859	KJ501990
		Ec16074	KJ721582
		C18219	KJ721583

Supplementary Table S5. Sequencing coverage of the seven assembled chloroplast genomes.

Species	Total covered bases (bp)	Genome size (bp)	Coverage*
**Vanilloideae**			
Vanilla planifolia	127134011	150127	847
**Cypripedioideae**			
Cypripedium formosanum	34236744	180723	189
Paphiopedilum armeniacum	30040152	165008	182
Paphiopedilum niveum	82923695	161387	514
**Orchidoideae**			
Goodyera fumata	149360589	157868	946
Habenaria longidenticulata	126061558	156152	807
**Epidendroideae**			
Masdevallia picturata	158689021	158276	1003

*: The coverage was computed by BWA-aligning reads back to each chloroplast genome, summing the number of reads covered at each base using SAMtools, and dividing them by the genome size.

Supplementary Table S6. Summary of the transcriptome, chloroplast and mitochondrial genome sequences of *Vanilla planifolia* (*ndh* deleted), *Paphiopedilum armeniacum* (*ndh* deleted), *Cypripedium formosanum* (*ndh* complete), *Habenaria longidenticulata*, *Goodyera fumata*, *Erycina pusilla* (*ndh* deleted) and *Masdevallia picturata* (*ndh* complete).

ndh gene	F	D	E	G	I	A	H	J	K	C	B
**Vanilloideae**											
Vanilla planifolia cDNA	-	-	-	-	-	-	-	25	15	44	8
Vanilla planifolia cp	-	-	-	-	-	-	-	-	-	-	19
Vanilla planifolia mt	-	-	59	-	-	-	-	81	46	69	99
**Cypripedioideae**											
Paphiopedilum armeniacum cDNA	-	30	-	-	-	6	80	100	100	100	62
Paphiopedilum armeniacum cp	-	78	-	-	-	-	-	100	100	100	100
Paphiopedilum armeniacum mt	55	78	60	-	-	60	98	-	-	78	85
Cypripedium singchii cDNA	7	94	100	94	83	8	74	98	98	100	65
Cypripedium formosanum cp	100	100	100	100	100	100	100	100	100	100	100
Cypripedium formosanum mt	6	-	-	-	-	-	-	-	-	58	-
**Orchidoideae**											
Habenaria delavayi	50	84	100	100	100	88	58	16	89	100	96
Habenaria longidenticulata cp	100	100	100	100	100	100	100	100	100	100	100
Habenaria longidenticulata mt	64	62	86	-	-	39	-	-	-	-	-
Goodyera pubescens cDNA	50	80	100	100	94	91	100	100	91	100	16
Goodyera fumata cp	100	100	100	100	100	100	100	100	100	100	100
Goodyera fumata mt	-	36	100	-	99	54	8	82	51	77	6
**Epidendroideae**											
Masdevallia yuangensis cDNA	67	79	82	72	68	44	88	100	100	100	100
Masdevallia picturata cp	100	100	100	100	100	100	100	100	100	100	100
Masdevallia picturata mt	-	99	100	100	98	93	99			30	84
Erycina pusilla cDNA	-	22	-	44	-	-	-	44	-	91	26
Erycina pusilla cp	-	60	-	52	-	-	32	42	-	68	43
Erycina pusilla mt	66	81	83	99	27	91	93	44		91	52

The numbers indicate the percentage of the nucleotide fragments that are similar to the *ndh* sequences. Red numbers indicate that the *ndh* DNA is in frame and encodes the full-length *ndh* gene.

Supplemental Table S7. Mitochondrial-derived *ndh* transcripts.

ndh	**Species**	**Transcript ID**	**Reference**
C	*Vanilla planifolia*	scaffold-THDM-2006247	Johnson et al. 2012
K	*Vanilla shenzhenica*	Unigene123_Va_fb	Tsai et al. 2013
C	*Phalaenopsis equestris*	Unigene17537_Pa	Tsai et al. 2013
C	*Erycina pusilla*	Unigene18133	Chou et al. 2013
J	*Erycina pusilla*	Unigene18740	Chou et al. 2013
D	*Erycina pusilla*	Unigene48484	Chou et al. 2013
B	*Vanilla planifolia*	scaffold-THDM-2032069	Johnson et al. 2012

Supplementary Table S8. Summary of 68 cp-like mt gene fragments.

Gene name	BAC clone ID	Position start	Position end	Coverage	RNA	Protein	Note
rps12	KJ501975	9650	9767	26/123	KJ501980		
	KJ501994	44996	44766	76/123	-		
psbA	KJ501968	17868	16944	334/353	-		
matK	-			0/518	-		
rps16	KJ501964	4875	5934	87/92	KJ501981		
	KJ501973	25866	24807	87/92	KJ501981		
psbK	KJ501970	15664	15483	61/61	-		
psbI	KJ501970	15247	15140	36/36	?	+	= cp
atpA	-			0/509	-		
atpF	-			0/184			
atpH	KJ501970	10590	10781	64/81	-		
atpI	KJ501970	9127	9867	247/247	-		*
rps2	KJ501970	43441	44148	236/236	-	+	
		8463	8828	118/236	-		
rpoC2	KJ501991	48917	49056	47/1385	-		
	KJ501970	39717	43208	1238/1385	-		
		6979	8396	504/1385	-		
rpoC1	KJ501991	46157	48720	680/680	-		indel
rpoB	KJ501991	43024	46127	1070/1070	-		indel
petN	KJ501991	40985	40899	29/29	-	+	
psbM	KJ501991	40363	40464	34/34	-	-	*
psbD	KJ501966	55970	56409	167/353	-		
	KJ501970	22390	22817	163/353	-		
	KJ501969	18498	18982	179/353	-		
	KJ501978	20972	21456	179/353	-		
psbC	KJ501969	19130	20279	407/407	-		indel
	KJ501978	21604	22753	407/407	-		indel
psbZ	KJ501969	20731	20907	62/62	-		*
	KJ501978	23205	23381	62/62	-		*
rps14	KJ501969	21840	21562	96/100	KJ501982		*
	KJ501978	24314	24036	96/100	KJ501982		*
psaB	KJ501969	23734	21916	606/734	-		
	KJ501978	26208	24390	606/734	-		
psaA	KJ501969	25073	23784	430/750	--		
	KJ501978	27547	26258	430/750	--		
ycf3	-			0/168			
rps4	-			0/201			
atpE	KJ501972	13239	13637	133/133	-	+	
	KJ501971	17639	17241	133/133	-	+	
	KJ501991	13373	13771	133/133	-	+	
	KJ501994	69656	69258	133/133	-	+	
atpB	KJ501972	11781	13222	489/497	--		indel
	KJ501971	19097	17656	489/497	-		indel
	KJ501991	11915	13356	489/497	-		indel
	KJ501994	71114	69673	489/497	-		indel
rbcL	KJ501972	11037	9646	464/480	KJ501983		indel
	KJ501971	19841	21232	464/480	KJ501983		indel
	KJ501991	11171	9780	464/480	KJ501983		indel
	KJ501994	71858	73249	464/480	KJ501983		indel
accD	KJ501972	8962	7563	488/488	KJ501984		indel
	KJ501971	21916	23315	488/488	KJ501984		indel
	KJ501991	9096	7697	488/488	KJ501984		indel
	KJ501994	73933	75332	488/488	KJ501984		indel
psaI	-			0/36	-		
ycf4	KJ501972	6510	5959	184/184	-		*
	KJ501971	24368	24919	184/184	-		*
	KJ501991	6644	6093	184/184	-		*

	KJ501994	76385	76936	184/184	-		*
cemA	KJ501972	2160	1483	229/229	KJ721582		*
	KJ501971	28718	29395	229/229	KJ721582		*
	KJ501991	2294	1617	229/229	KJ721582		*
	KJ501994	80735	81412	229/229	KJ721582		*
petA	-			0/320	-		
psbJ	-			0/40	-		
psbL	KJ501976	24797	24887	33/38	KJ501986		
psbF	KJ501976	24655	24771	39/39	KJ501986		*
psbE	KJ501976	24421	24636	78/83	-		
petL	KJ501973	20048	20140	31/31	KJ501987		*
petG	KJ501973	20325	20435	37/37	KJ501987		*
psaJ	KJ501975	12331	12212	44/44	KJ501988	+	
rpl33	KJ501975	11746	11549	66/66	-		*
rps18	KJ501975	11362	11051	104/104	KJ501980		*
rpl20	KJ501975	10408	10717	103/120	KJ501980		indel
rps12	KJ501994	44996	44766	77/123	-		
	KJ501975	9650	9767	38/123	KJ501980		
clpP	KJ501975	7537	9533	202/202	KJ501980		indel
psbB	KJ501975	6964	5509	508/508	-		indel
psbT	KJ501975	5170	5066	35/35	-		*
psbN	KJ501975	4915	5043	43/43	-		*
psbH	KJ501975	4803	4585	73/73	-	+	
petB	KJ501975	3684	3071	214/217	-		
petD	KJ501968	18529	18096	148/163	-		
rpoA	KJ501976	23700	22817	315/339	KJ501986		
rps11	KJ501976	24099	23777	136/138	KJ501986		
rpl36	KJ501976	24351	24241	35/35	KJ501986	+	
infA	KJ501973	2763	2912	49/77	-		
		24316	24414	32/77	KJ501981		
	KJ501964	6425	6327	32/77	KJ501981		
rps8	KJ501964	7376	7007	131/131	KJ501981		indel
	KJ501973	2287	2637	122/131	KJ501981		
		24149	24196	16/131	-		
rpl14	KJ501973	1744	2109	122/122	KJ501981		
	KJ501964	7919	7554	122/122	KJ501981		
rpl16	KJ501973	1219	1611	132/136	KJ501981		
	KJ501964	8444	8052	132/136	KJ501981		
rps3	KJ501973	46223	46399	59/219	KJ501990		
		23730	24102	137/219	KJ501990		
	KJ501964	10641	10075	219/219	KJ501990		indel
rpl22	KJ501973	11059	10697	121/121	KJ501990	+	
	KJ501964	45805	46167	121/121	KJ501990	+	
rps19	KJ501973	45343	45618	91/92	KJ501990		
	KJ501964	11521	11246	91/92	KJ501990		
rpl2	KJ501973	44668	45099	143/272	KJ501990		
	KJ501964	12196	11765	143/272	KJ501990		
rpl23	KJ501968	9239	9469	77/93	KJ721583		
ycf2	KJ501994	34804	41254	2225/2225	-		indel
rps7	KJ501994	44150	43707	154/155	-		
rpl32	KJ501978	10929	10774	52/56	-		
	KJ501969	8455	8300	52/56	-		
ccsA	KJ501975	12929	13805	326/326	KJ501988		indel
psaC	KJ501978	9344	9586	81/81	-	+	
	KJ501969	6870	7112	81/81	-	+	
rps15	KJ501978	3922	4191	90/90	-	-	*
	KJ501969	1448	1717	90/90	-	-	*

Supplementary Table S9. Mitochondria and chloroplast genome accession numbers in identifying the *ndh* genes and cp-like DNA from the published plant mt genomes.

Species	mt DNA accession	cp DNA accession
Amborella trichopoda	KF754803.1	NC_005086.1
Anomodon rugelii	NC_016121.1	-
Arabidopsis thaliana	NC_001284.2	NC_000932.1
Bambusa oldhamii	EU365401.1	FJ970915.1
Beta macrocarpa	NC_015994.1	EF534108.1 (*Beta vulgaris*)*
Beta vulgaris	BA000024.1	EF534108.1
Boea hygrometrica	NC_016741.1	NC_016468.1
Brassica carinata	NC_016120.1	NC_016734.1 (*Brassica napus*)
Brassica juncea	NC_016123.1	NC_016734.1 (*Brassica napus*)
Brassica napus	NC_008285.1	NC_016734.1
Brassica oleracea	NC_016118.1	NC_016734.1 (*Brassica napus*)
Carica papaya	NC_012116.1	EU431223.1
Chlorokybus atmophyticus	NC_009630.1	DQ422812.2
Citrullus lanatus	NC_014043.1	DQ865976.1 (*Cucumis sativus*)
Cucumis melo	JF412792.1	DQ865976.1 (*Cucumis sativus*)
Cucumis sativus	HQ860792.1	DQ865976.1
Cucurbita pepo	NC_014050.1	DQ865976.1 (*Cucumis sativus*)
Cycas taitungensis	NC_010303.1	NC_009618.1
*Cymbidium sinense**		NC_021430.1
Cypripedium formosanum		KJ501998
Daucus carota	NC_017855.1	NC_008325.1
Erycina pusilla		NC_018114.1
Goodyera fumata		KJ501999
Habenaria longidenticulata		KJ524104
Lotus japonicus	NC_016743.2	NC_002694.1
Marchantia polymorpha	NC_001660.1	NC_001319.1
Masdevallia picturata		KJ566305
Megaceros aenigmaticus	NC_012651.1	-
Millettia pinnata	NC_016742.1	NC_016708.2
Nicotiana tabacum	NC_006581.1	Z00044.2
Oncidium 'Gower Ramsey'		NC_014056.1
Oryza rufipogon	NC_013816.1	AY522330.1 (*Oryza sativa* subsp. *japonica*)
Oryza sativa subsp. *indica*	NC_007886.1	AY522330.1 (*Oryza sativa* subsp. *japonica*)
Oryza sativa subsp. *japonica*	NC_011033.1	AY522330.1
Paphiopedilum armeniacum		KJ566307
Paphiopedilum niveum		KJ524105
Phaeoceros laevis	NC_013765.1	-
Phoenix dactylifera	JN375330.1	GU811709.2
Physcomitrella patens	NC_007945.1	NC_005087.1
Pleurozia purpurea	NC_013444.1	-
Ricinus communis	NC_015141.1	NC_016736.1
Silene latifolia	NC_014487.1	NC_016730.1
Silene vulgaris	JF750427.1	NC_016727.1
Sorghum bicolor	NC_008360.1	EF115542.1
Spirodela polyrhiza	NC_017840.1	NC_015891.1
Tripsacum dactyloides	NC_008362.1	EF115542.1 (*Sorghum bicolor*)
Triticum aestivum	NC_007579.1	AB042240.3
Vanilla planifolia		KJ566306
Vigna radiate	NC_015121.1	NC_013843.1
Vitis vinifera	NC_012119.1	DQ424856.1
Zea luxurians	NC_008333.1	AY928077.1 (*Zea mays*)
Zea mays	NC_007982.1	AY928077.1
Zea perennis	NC_008331.1	AY928077.1 (*Zea mays*)

- Unavailable

* indicates there is no mt genome available. The mt genome of the species in parenthesis is used for further analysis

Supplementary Table S10. Primer list for BAC clone screening.

**For BAC clone screening**			
Sequence ID	Forward	Reverse	Clone
Ep-mt-circle1-175J16	TGGGGCATTAGTGAAAGGAT	AAGCCTGCCAGCAAGCATT	175J16
Ep-mt-circle2_162I22	TCGCTATTTGATTTCATCCT	GCACGAAAAAGTAACCAGAC	162I22
Ep-mt-*ndhJC*_002O08	TGCGCGGTACAAAGTTCA	CCAGGCGAATTGTTAGGC	002O08
Ep-mt-cp1_002O08	GCCATGCAACCGGATAAG	TAGCTCCGGGTCCAGAAA	002O08
Ep-mt-*ndhC*_180K03	TATATATGCCGGATGCTCTT	TTATTCGGAGAGTCACCTTG	180K03
Ep-mt-cp2_163A04	TCGAGCTACCGCCCTATG	TTGGCCGTAGAAGGATCG	163A04
Ep-mt-*ndhD*_162P19	TGATCCATTGCTAACATCAA	TTTGTTTGTCTTTCTGGTCC	162P19
Ep-mt-cp3_123E16	TTTGCACTTTCATTCCTTTT	GTCGGAGGAGGTTATTTCTT	123E16
Ep-mt-cp4_092M20	AGCAAGCTAACCTCTCGCTTT	TAAGTACCATTGCAGCGCCTA	092M20
Ep-mt-*ndhB*_089F15	CAGACCTTTCCTCTTTTGTG	GAAACGAAGTGGATAACAGC	089F15
Ep-mt-cp5_041C10	AGAAACGCGAGGGAATCA	TGGGGTGAGTTTGATTCAAGT	041C10
Ep-mt-cp5_037I23	TAGTGGGAGGTCGCGAAG	TCCCCCACCCCAAGTATT	037I23
Ep-mt-*ndhDH*_037D12	AAGTGACGCACTCTTTCATT	GAGGCACCATTTGTGACTAT	037D12
Ep-mt-cp6_034M07	GGAGGGAGAAGCGGAGTC	CAGGGAGGTTCGGGAAGT	034M07
Ep-mt-cp7_024M14	TGAAAATGCTCCTTTCTCAT	GACATCGCTAGTCATCCATT	024M14
Ep-mt-*ndhDF*_015H07	AGAAACTGCAGTCGTTAAGC	TTTGCTCTGATTAGGAGGAG	015H17
Ep-mt-cp8_012N23	GGAGAGCAGCTTCTTGAGTA	GTACTGAACGGATTGGAATG	012N23

**For circle form demonstration**			
Sequence ID	Forward	Reverse	Clone
Ep-mt-cp8_012N23	GAGGCAGGTGAGAGTCTTGG	ACGCAAAGAGAGGTCGTGAT	012N23
Ep-mt-*ndhDH*_037D12	TCCAGCTTTGAGGAAGCAAT	TCAGCCCATGAGTCAGTGTC	037D12
Ep-mt-cp3_123E16	CGGTCACAAATTGGGAGAAT	CATCCTTCCCTTTTTCCACA	123E16
Ep-mt-circle2_162I22	TCACTGATGGATTCGGTCAA	AGTCACTCTCGACGGGAGAA	162I22
Ep-mt-cp2_163A04	AGCCTTAGTGCACCAACCAC	TTAGGGCGAAACTGCCTATG	163A04
Ep-mt-circle1-175J16	GGACCGTCAACTCCAACTGT	TGGATAGGAAGAGGCATTGG	175J16

Supplemental Figure S1. Gene maps of orchid chloroplast genomes.

Genes on the outside of the map are transcribed clockwise whereas genes on the inside of the map are transcribed counterclockwise. Colors indicate genes with different functional groups. *Paphiopedilum niveum* and *Paphiopedilum armeniacum* have the same genome structure

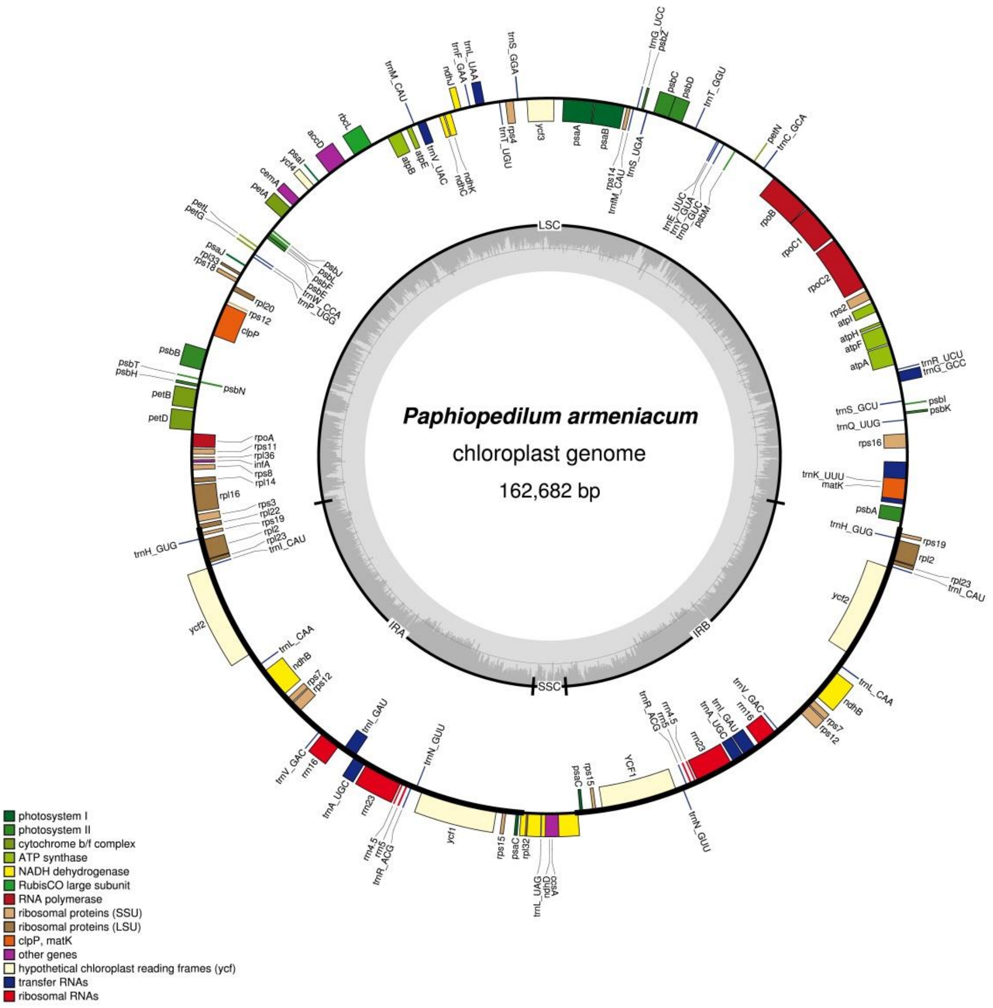

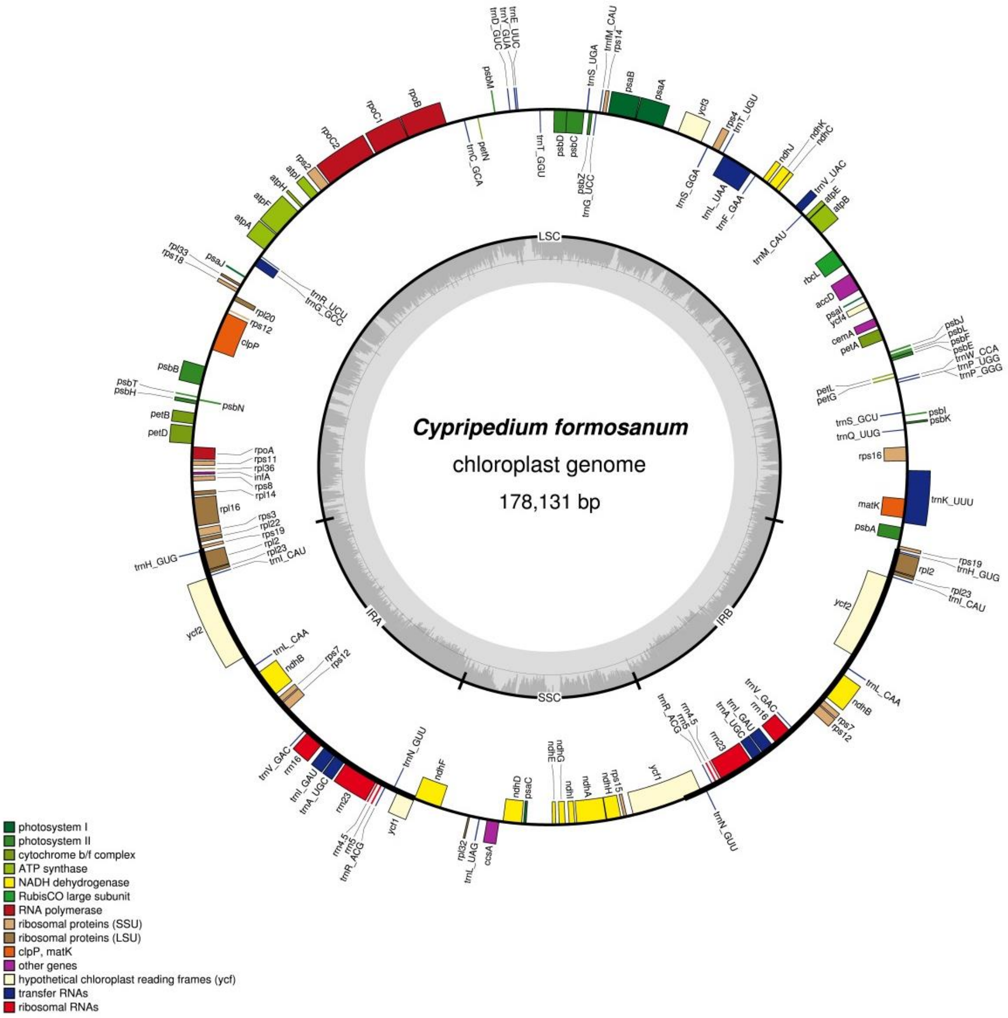
Cypripedium formosanum
chloroplast genome
178,131 bp
LSC
SSC
IRA
IRB
photosystem I
photosystem II
cytochrome b/f complex
ATP synthase
NADH dehydrogenase
RubisCO large subunit
RNA polymerase
ribosomal proteins (SSU)
ribosomal proteins (LSU)
clpP, matK
other genes
hypothetical chloroplast reading frames (ycf)
transfer RNAs
ribosomal RNAs

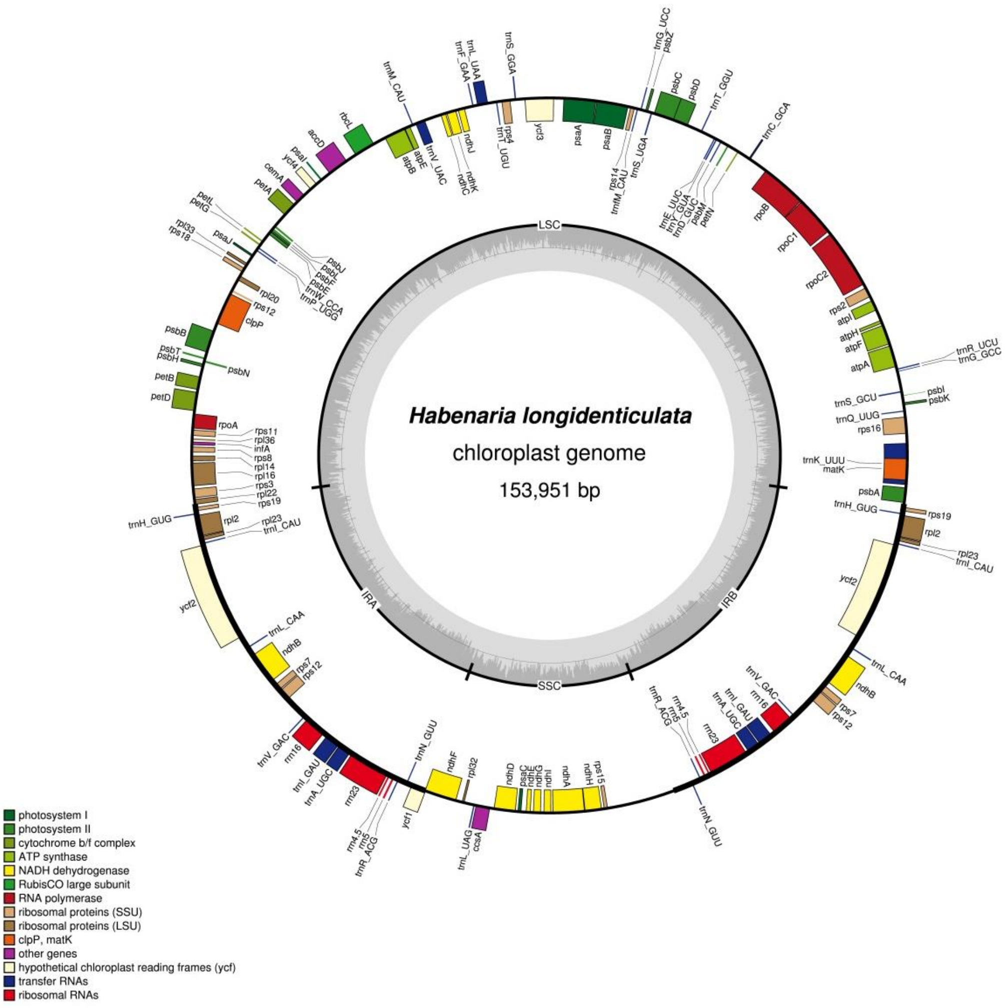
Habenaria longidenticulata
chloroplast genome
153,951 bp
LSC
SSC
IRA
IRB
photosystem I
photosystem II
cytochrome b/f complex
ATP synthase
NADH dehydrogenase
RubisCO large subunit
RNA polymerase
ribosomal proteins (SSU)
ribosomal proteins (LSU)
clpP, matK
other genes
hypothetical chloroplast reading frames (ycf)
transfer RNAs
ribosomal RNAs

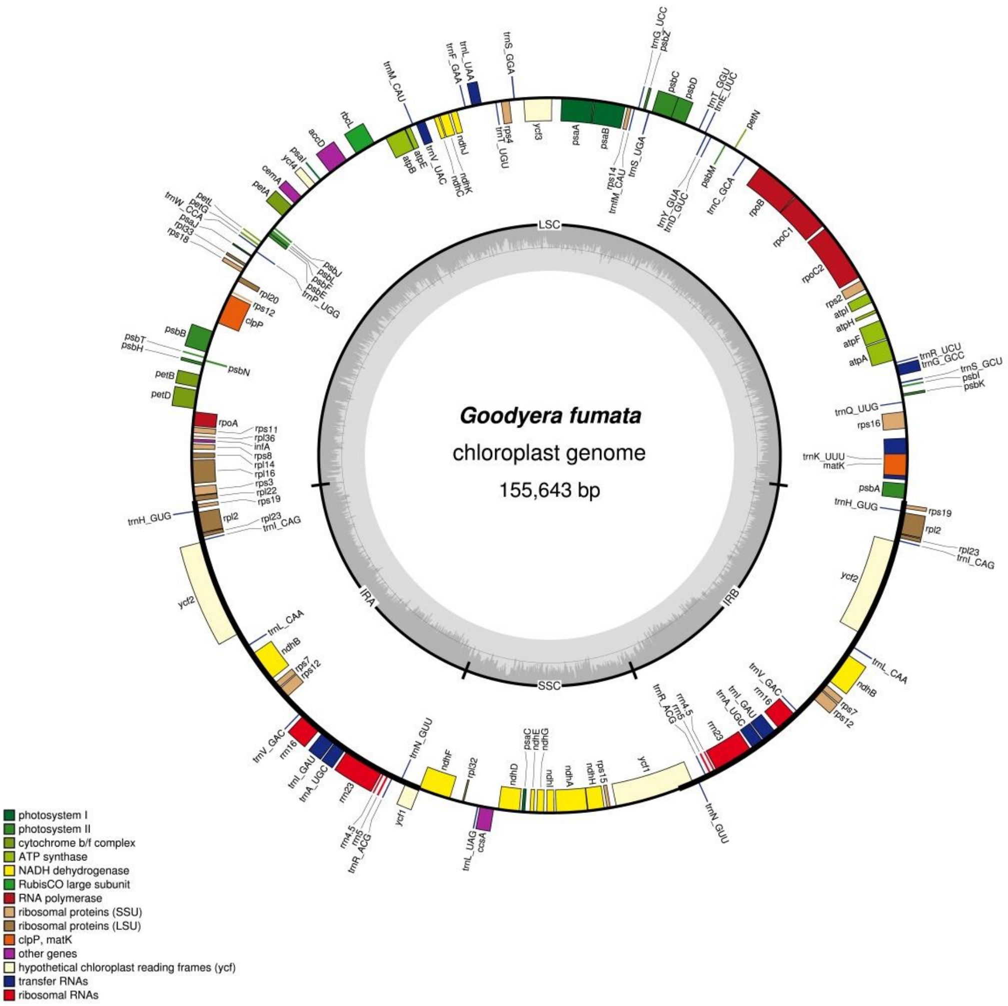

Goodyera fumata
chloroplast genome
155,643 bp
LSC
SSC
IRA
IRB
photosystem I
photosystem II
cytochrome b/f complex
ATP synthase
NADH dehydrogenase
RubisCO large subunit
RNA polymerase
ribosomal proteins (SSU)
ribosomal proteins (LSU)
clpP, matK
other genes
hypothetical chloroplast reading frames (ycf)
transfer RNAs
ribosomal RNAs

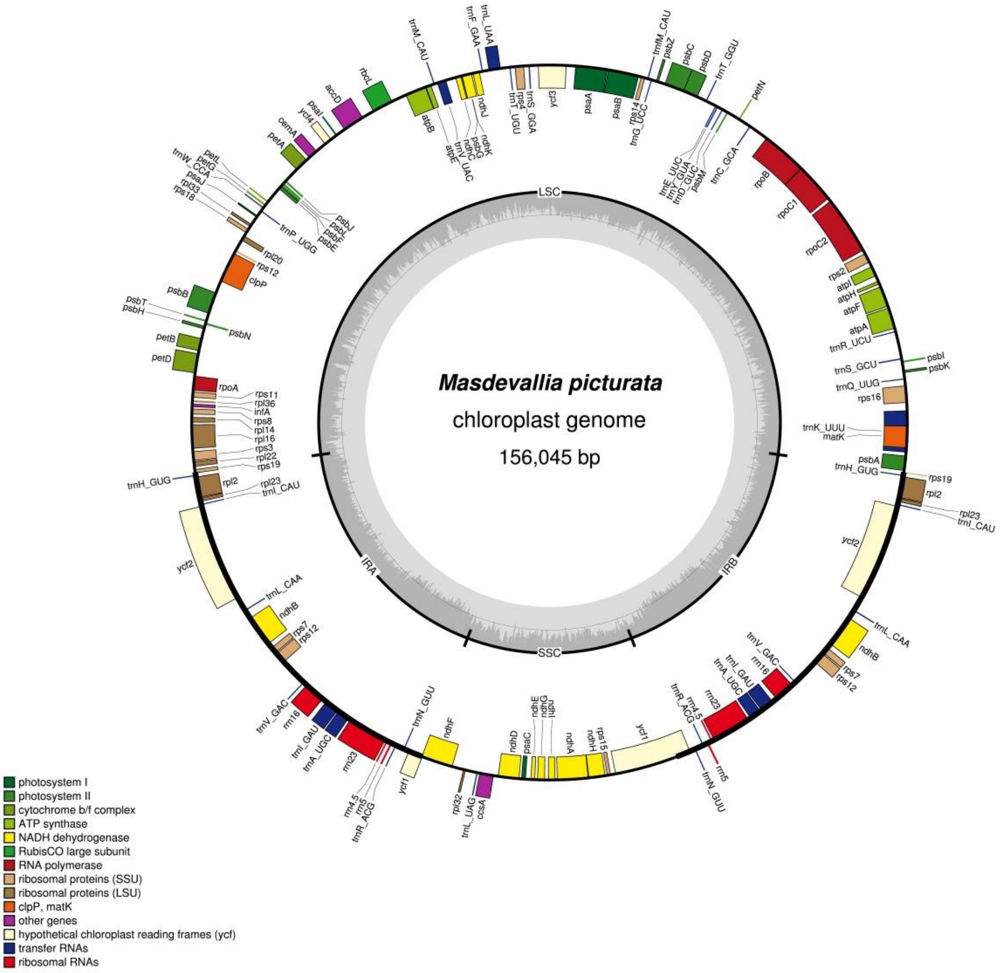

Masdevallia picturata
chloroplast genome
156,045 bp
LSC
SSC
IRA
IRB
photosystem I
photosystem II
cytochrome b/f complex
ATP synthase
NADH dehydrogenase
RubisCO large subunit
RNA polymerase
ribosomal proteins (SSU)
ribosomal proteins (LSU)
clpP, matK
other genes
hypothetical chloroplast reading frames (ycf)
transfer RNAs
ribosomal RNAs

Supplementary Figure S2. The cp *ndhJ-K-C* genes were transferred to the plant mt genome. The boxes indicate the putative coding regions of the genes. The arrowheads indicate the direction of the genes. The accession no. of the sequences are listed in Supplementary Table S4.

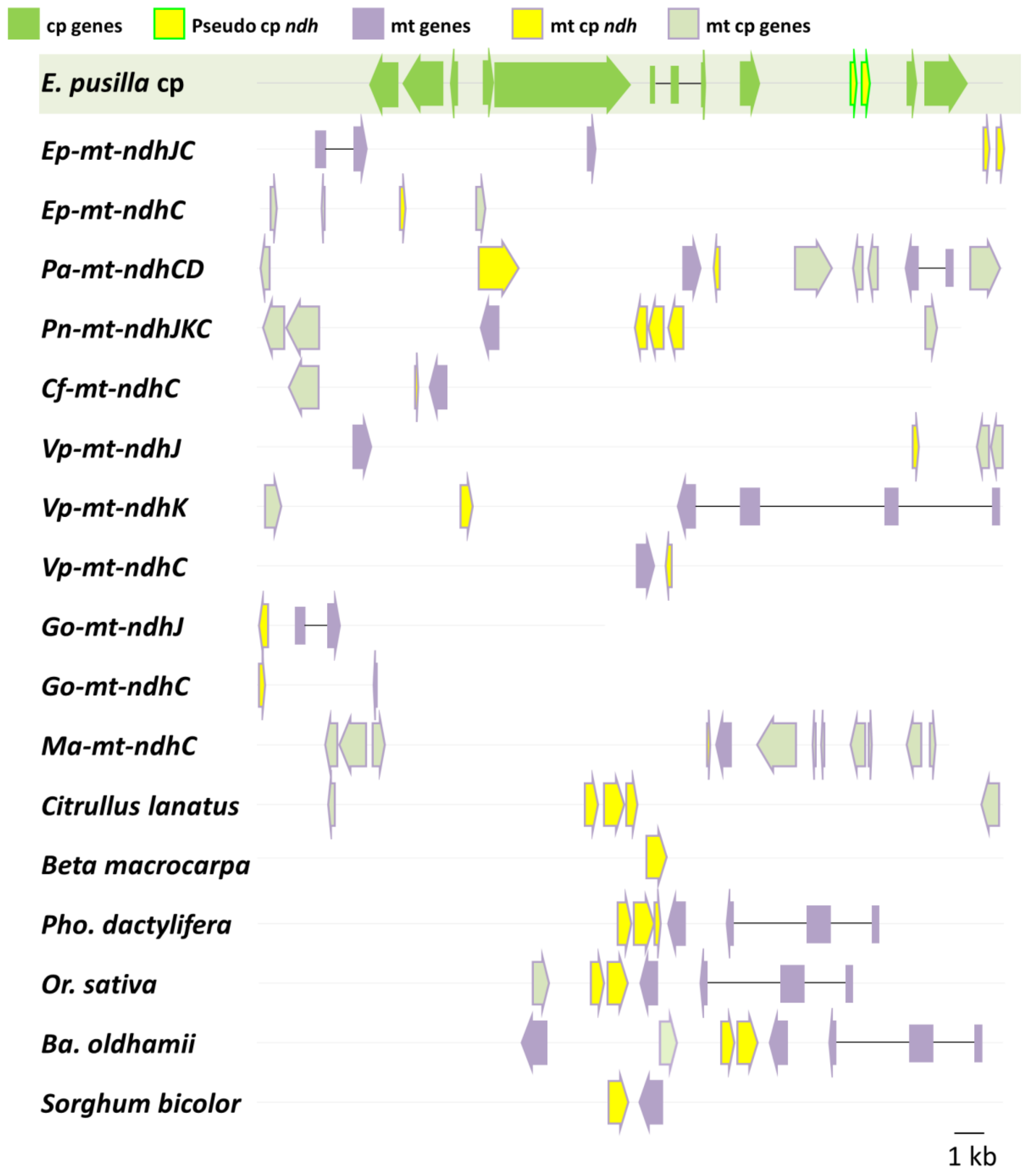

Supplementary Figure S3. The cp *ndhF-D-E-G-I-A-H* region was transferred to the plant mt genome. The boxes indicate the putative coding regions of the genes. The arrowheads indicate the direction of the genes. The accession no. of the sequences are listed in Supplementary Table S4.

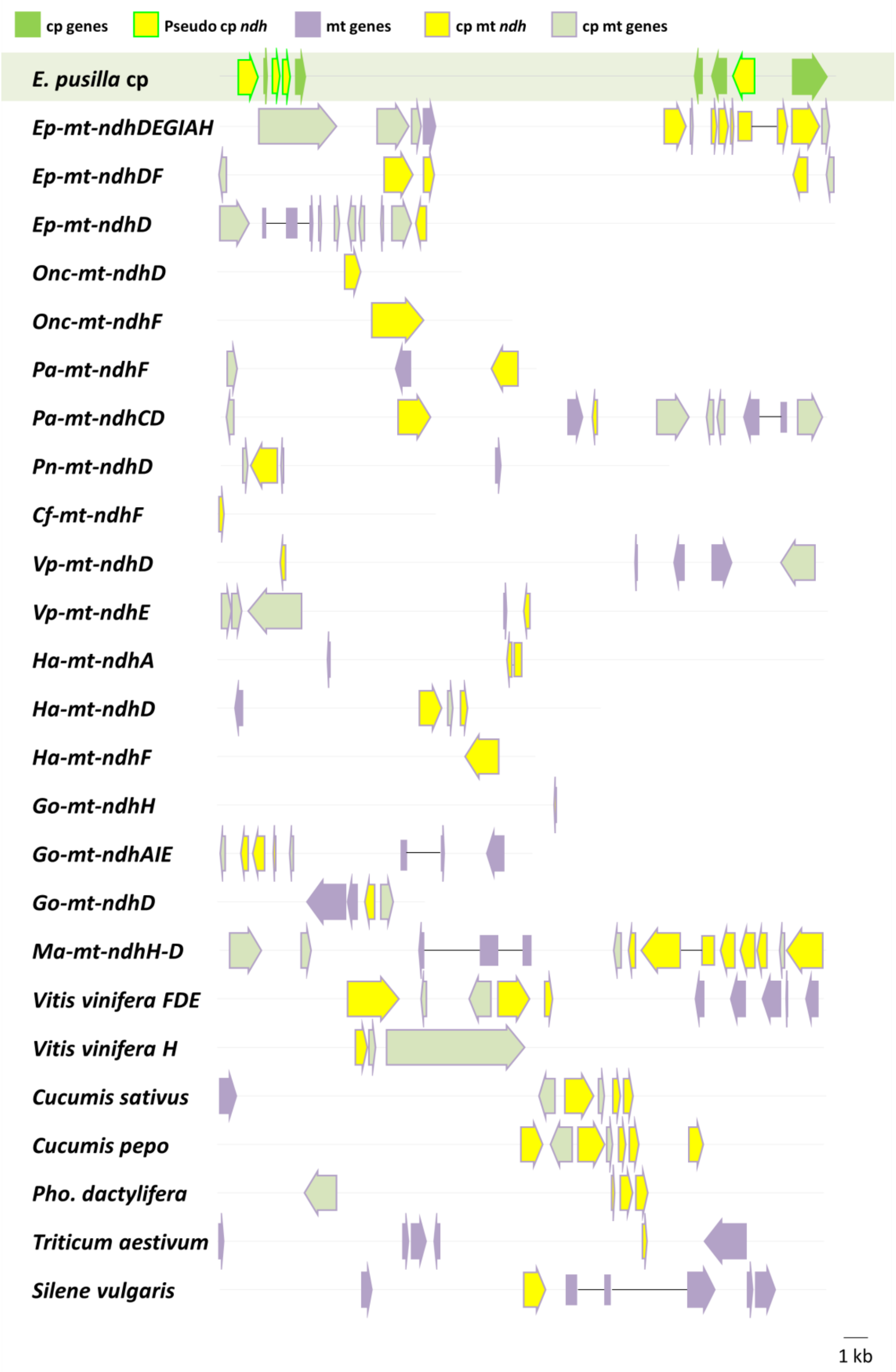

Supplementary Figure S4. The cp *ndhB* gene fragment was transferred to the plant mt genome. The boxes indicate the putative coding regions of the genes. The arrowheads indicate the direction of the genes. The accession no. of the sequences are listed in Supplementary Table S4.

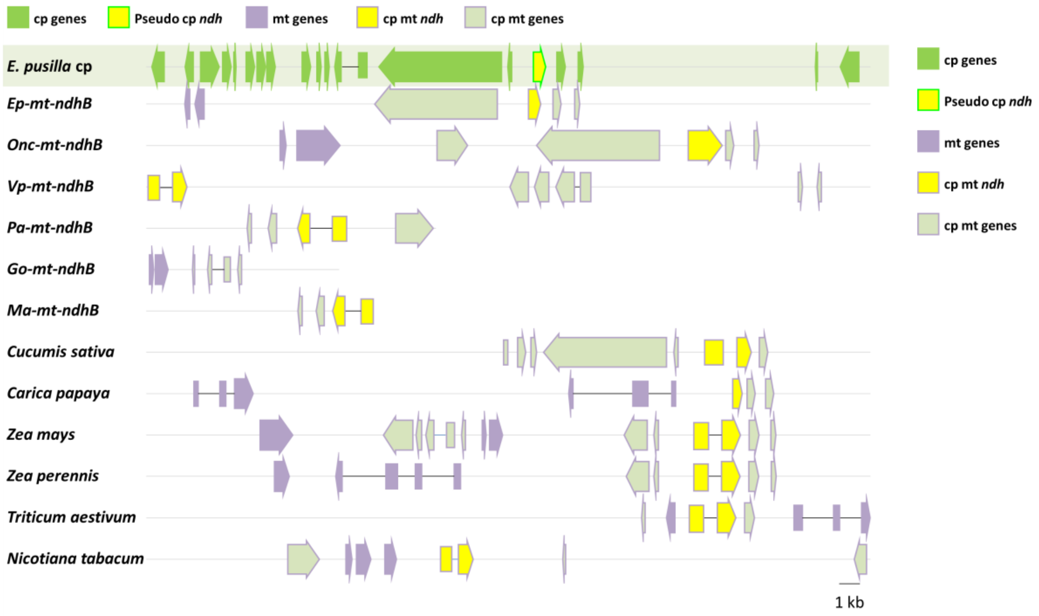

Supplementary Figure S5. Flow chart of sequence annotation.

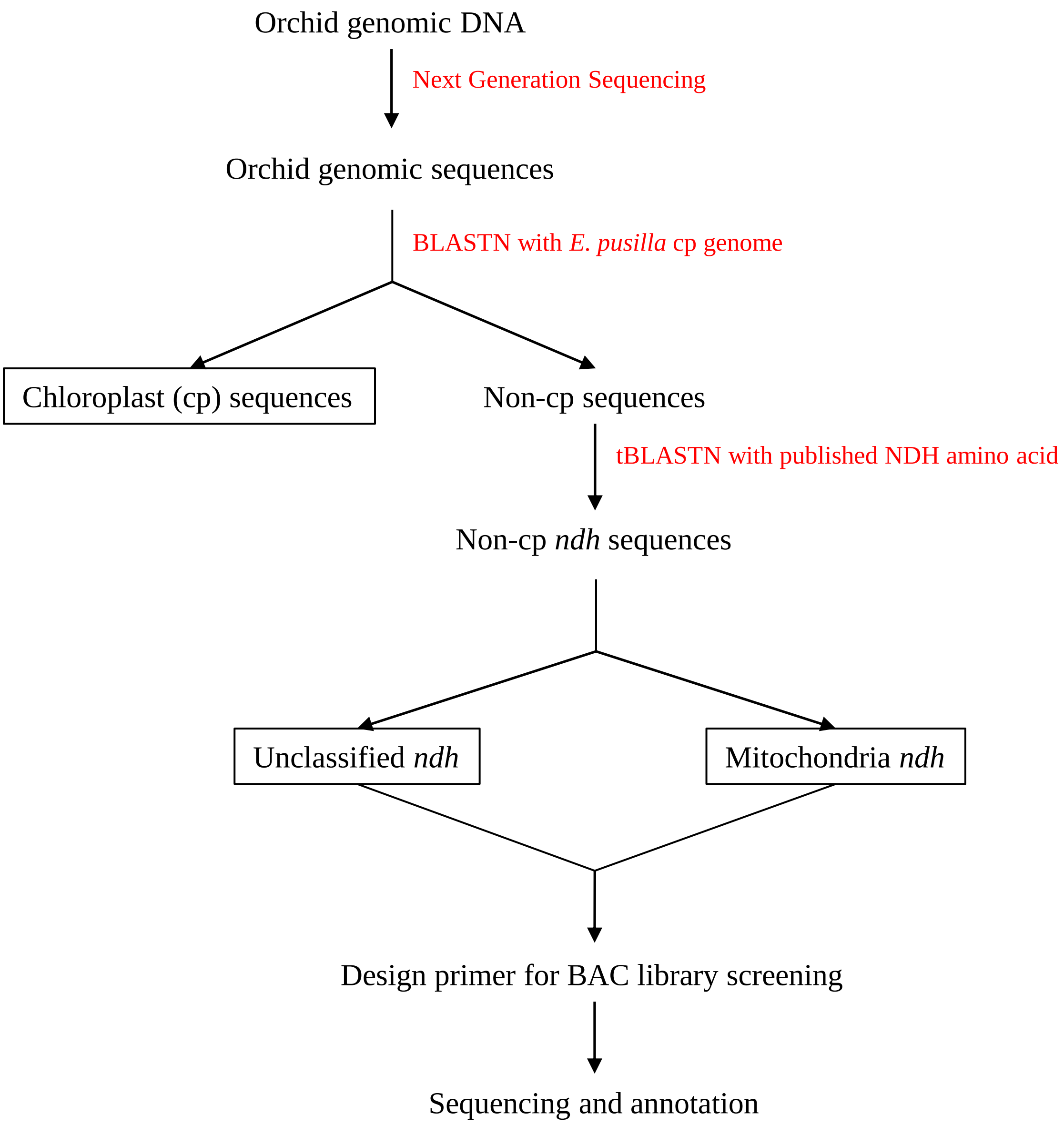

Supplement: Supplementary Information [file srep09040-s1.pdf]
